# TensoGraph: a tensor–transformer framework for global–local drug synergy prediction on heterogeneous graphs

**DOI:** 10.1093/bib/bbag157

**Published:** 2026-04-14

**Authors:** Xi Wang, Dongxue Zhang, Limin Li

**Affiliations:** School of Mathematics and Statistics, Xi’an Jiaotong University, Xianning West 28, Xi’an 710049, Shaanxi, China; School of Mathematics and Statistics, Xi’an Jiaotong University, Xianning West 28, Xi’an 710049, Shaanxi, China; School of Mathematics and Statistics, Xi’an Jiaotong University, Xianning West 28, Xi’an 710049, Shaanxi, China

**Keywords:** drug combination, attention mechanism, synergistic effect, heterogeneous graph, tensor decomposition

## Abstract

Combination therapy, which administers multiple drugs either simultaneously or sequentially, aims to enhance therapeutic efficacy while minimizing side effects and drug resistance common in monotherapies for complex diseases. Identifying drug combinations that exhibit synergistic effects is therefore critical to both biomedical research and clinical practice. In this study, we introduce TensoGraph, a novel tensor–transformer model that integrates gene expression, drug structure, and physiochemical fingerprints within heterogeneous graphs to capture global–local interactions for drug synergy prediction. Specifically, we construct cell line-specific heterogeneous drug combination graphs where edges represent synergistic, additive, or antagonistic interactions. To capture global patterns, we apply Tucker tensor decomposition on the multi-relational interaction tensor to extract low-dimensional drug embeddings that reflect holistic interaction profiles. Concurrently, a heterogeneous graph transformer network is employed to learn local structural representations via automatic meta-path discovery. The global and local features are further integrated with drug molecular structure and physicochemical fingerprints to construct comprehensive drug representations. Extensive experiments on multiple benchmark datasets demonstrate that TensoGraph significantly outperforms state-of-the-art baselines in predicting synergistic drug combinations, with improved biological interpretability. These results underscore the effectiveness of tensor-based global–local modeling for capturing complex drug interaction mechanisms and facilitating the discovery of personalized combination therapies.

## Introduction

Combination therapy, which administers two or more drugs either simultaneously or sequentially, is a widely adopted strategy to enhance treatment efficacy while mitigating side effects and delaying resistance development [1, 2]. This approach has demonstrated particular promise in complex diseases such as cancer [3, 4] and hypertension [5]. By reducing dose-dependent toxicity [6] and curbing the evolution of resistance in pathogens or tumor cells [7], combination therapy provides clear advantages over monotherapy. However, drug combinations may yield synergistic effects that exceed additive expectations, additive effects that reflect independent contributions, or antagonistic effects that diminish therapeutic outcomes [3, 8, 9]. Therefore, identifying synergistic drug combinations tailored to specific cellular contexts is a fundamental challenge in both clinical and computational pharmacology.

Traditional identification of synergistic drug pairs has relied heavily on clinical trials and high-throughput screening, which are resource-intensive, time-consuming, and potentially risky to patients [10, 11]. As the number of available drugs continues to grow, exhaustively exploring all possible pairs becomes computationally and experimentally infeasible. Consequently, there is an urgent need for computational approaches that can efficiently and accurately predict synergistic drug combinations.

Recent advances in machine learning and deep learning have significantly accelerated research in drug synergy prediction. Early approaches adopted conventional machine learning models such as random forests, logistic regression, and XGBoost [12–15], leveraging handcrafted features derived from drug fingerprints and cell line gene expression profiles. The introduction of *DeepSynergy* [16] marked the beginning of deep learning-based models, employing fully connected neural networks to model nonlinear relationships between drug and cell line features.

To improve structural understanding of drug compounds, later models integrated graph-based molecular representations. *MatchMaker* [17] employed graph convolutional networks (GCNs) to learn structural embeddings from molecular graphs. *DeepDDS* [18] introduced attention mechanisms for capturing pairwise interactions, and *PRODeepSyn* [19] further incorporated protein–protein interaction networks to model indirect biological relationships. Multi-modal fusion strategies, such as those used in *TranSynergy* [20], utilized transformers to model higher order dependencies across modalities.

Despite these advances, existing methods often fail to model the heterogeneous nature of drug interactions. In reality, drugs may interact synergistically, additively, or antagonistically depending on the cellular context, and this interaction is inherently multi-relational. Meanwhile, emerging tensor-based methods such as *TuckER* [21] and *DeepTensor* [22] demonstrate the potential of tensor decomposition in capturing global, high-order relational patterns in multimodal biomedical data. However, these approaches have yet to be fully integrated with heterogeneous graph learning frameworks in the context of drug synergy.

To address these limitations, we propose **TensoGraph**, a novel framework for global-local drug synergy prediction that integrates tensor decomposition and heterogeneous graph transformers. For each cell line, we construct a heterogeneous drug combination graph in which nodes represent drugs and edges encode three types of interactions: synergistic, additive, and antagonistic. Tucker decomposition is employed on the resulting three-mode tensor to extract **global interactive features** across all drug combinations. In parallel, we adopt a graph transformer network (GTN) to model **local interactive features** by dynamically learning meta-paths within cell line-specific graphs. These global and local features are then fused with structural embeddings derived from drug molecular graphs and physicochemical fingerprints to form comprehensive drug representations.

Extensive experiments on benchmark datasets demonstrate that *TensoGraph* consistently outperforms state-of-the-art baselines in synergy prediction tasks. Moreover, ablation studies validate the effectiveness of integrating global and local features through tensor and heterogeneous graph learning. These results underscore the potential of TensoGraph to advance precision medicine by enabling efficient and interpretable prediction of synergistic drug combinations across diverse cellular environments.

## Methods

### Overview

**Figure 1:**
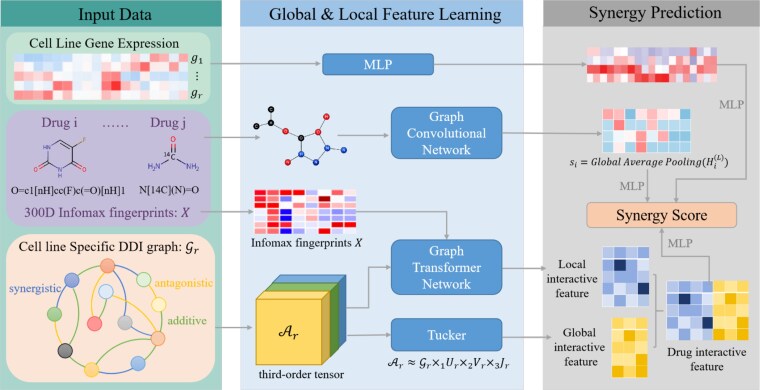
Framework of TensoGraph for cell line-specific drug synergy prediction. TensoGraph integrates multisource biological information to jointly capture global and local drug interaction features within specific cellular contexts. For each drug pair in a given cell line, drug molecular fingerprints and gene expression profiles are provided as inputs, together with a cell line-specific DDI graph. Drug fingerprints $X$ are combined with the DDI graph $\mathcal{A}_{r}$ and processed by a GTN to learn local interaction representations that reflect cell line-dependent relational patterns. In parallel, a Tucker tensor decomposition is applied to the DDI graph to extract global interaction features from the third-order tensor $\mathcal{A}_{r}$. These global and local embeddings, together with drug representations encoded by a GCN and cell line representations learned by an MLP, are integrated through a predictive module to generate the final synergy score, enabling accurate modeling of both molecular structure and context-specific pharmacological effects.

The primary objective of this study is to develop a prediction model that scores synergistic effects based on triples (drug $i$, drug $j$, cell line $r$). For the feature representation of drugs $i$ and $j$, we integrate molecular structural features derived from SMILES (Simplified Molecular Input Line Entry System) sequences and chemoinformatics-driven molecular fingerprint characteristics as foundational features. For cell line $r$, gene expression data are employed as the characteristic feature. A large-scale drug combination effect training dataset encompassing multiple cell lines was constructed, incorporating quantitative scores for synergistic effects, additive effects, and antagonistic effects. We propose a novel model, TensoGraph, that can predict drug–drug synergistic scores via integrating structures and physicochemical fingerprints of drugs, gene expression levels of cell lines, and the cell line-specific global and local interactive features among drugs. The overview of the framework is shown in Fig. 1. The main symbols are summarized in Table 1. For each cell line, we construct a cell line-specific drug combination heterogeneous graph comprising three distinct types of edges—synergistic, additive, and antagonistic—each quantitatively weighted by corresponding interaction scores to reflect the nature and strength of drug–drug interactions (DDIs). Based on the drug heterogeneous graph, our TensoGraph captures cell line-specific global drug interactive features via tensor decomposition-based latent factor modeling, and simultaneously integrates local relational patterns through a heterogeneous graph neural network that models the multidimensional relationships among drugs. This architecture enables in-depth mechanistic interpretation of drug combination synergism within cell line-specific microenvironments, ultimately achieving high-precision prediction of synergistic effect scores for novel drug combinations in targeted cell lines, thereby providing data-driven insights for optimizing combinatorial therapeutic strategies.

**Table 1:** Summary of mathematical symbols used in TensoGraph.

Symbol	Description
$i, j$	Indices of drugs in a drug pair
$r$	Index of a cell line
$n$	Number of drugs
$d$	Dimensionality of learned drug embeddings
$m$	Fingerprint feature dimension
$R$	Number of cell lines
$C$	Number of channels in the GTN
$L$	Number of GTN layers
$\boldsymbol{\mathcal{G}}_{r}$	Drug combination heterogeneous graph in cell line $r$
$\mathcal{V}$	Set of all drugs
$\mathbf{X}\in \mathbb{R}^{n\times m}$	Fingerprint feature of all drugs
$\mathbf{x}_{i}\in \mathbb{R}^{m}$	Fingerprint features of drug $i$
$\mathcal{E}_{r}$	Set of edges in cell line $r$
$\mathbf{A}_{r}\in \mathbb{R}^{n\times n}$	Adjacency matrix of all drugs interaction in $r$
$\mathcal{E}_{r,1}$	Synergistic interaction edges
$\mathcal{E}_{r,2}$	Additive interaction edges
$\mathcal{E}_{r,3}$	Antagonistic interaction edges
$\mathbf{A}_{r|1}\in \mathbb{R}^{n\times n}$	Adjacency matrix of the synergistic subgraph
$\mathbf{A}_{r|2}\in \mathbb{R}^{n\times n}$	Adjacency matrix of the additive subgraph
$\mathbf{A}_{r|3}\in \mathbb{R}^{n\times n}$	Adjacency matrix of the antagonistic subgraph
$\boldsymbol{\mathcal{A}}_{r} \in \mathbb{R}^{n\times n\times 3}$	Stacked interaction third-order tensors for $r$
$\mathbf{U}_{r}\in \mathbb{R}^{n\times R_{1}}$	Tucker-decomposed drug global feature in $r$
$\mathbf{Z}_{r}\in \mathbb{R}^{n\times d}$	Local drug interaction representation in $r$
$\mathbf{W}_{r}^{(l)}\in \mathbb{R}^{3\times C}$	Learnable edge-type weight at GTN layer $l$ in $r$
$\boldsymbol{\mathcal{A}}_{r}^{(l)}\in \mathbb{R}^{n \times n\times C}$	Learned composite adjacency matrix at GTN layer $l$
$\mathbf{A}_{c}^{(l)}\in \mathbb{R}^{n\times n}$	$c$ th channel of $\boldsymbol{\mathcal{A}}_{r}^{(l)}$
$\mathbf{W}_{r}\in \mathbb{R}^{m\times d}$	Learnable projection matrix for local features in $r$
$\mathbf{a}_{r}\in \mathbb{R}^{C}$	Channel-level attention scores
$\mathbf{H}_{r}$	Concatenated global–local drug representation
$\mathbf{h}_{r,i}$	Integrated interaction feature of drug $i$ in cell line $r$
$\mathbf{s}_{i}$	Molecular structure embeddings of drug $i$
$\mathbf{g}_{r}$	Gene expression feature vector of cell line $r$
$\mathbf{z}_{i,j,r}$	Concatenated feature for drug pair $(i,j)$ in $r$
$\hat{y}_{i,j,r}$	Predicted synergy score for drug pair $(i,j)$ in $r$
$y_{i,j,r}$	Ground-truth synergy score

### Cell line-specific DDI graph construction

We construct drug combination heterogeneous graphs for each cell line. For a given cell line $ r $, the drug combination graph is defined as a heterogeneous graph:


\begin{align*} & \boldsymbol{\mathcal{G}}_{r} = (\mathcal{V}, \mathcal{E}_{r}, \mathbf{X}, \mathbf{A}_{r}), \end{align*}

where $ \mathcal{V} $ denotes the set of drugs, $ \mathbf{X} \in \mathbb{R}^{n \times m} $ represents the physicochemical fingerprint matrix of drugs, and


\begin{align*} & \mathcal{E}_{r} = \mathcal{E}_{r,1} \cup \mathcal{E}_{r,2} \cup \mathcal{E}_{r,3} \end{align*}

is the union of three edge sets corresponding to synergistic ($ \mathcal{E}_{r,1} $), additive ($ \mathcal{E}_{r,2} $), and antagonistic ($ \mathcal{E}_{r,3} $) interactions. The adjacency matrix $ \mathbf{A}_{r} \in \mathbb{R}^{n \times n} $ encodes the interaction type between each drug pair, where


\begin{align*} & \mathbf{A}_{r}(i,j) \begin{cases} \mathbf{A}_{r|1}(i,j), & \mathrm{if}\ (i,j) \text{ exhibits synergistic interaction}, \\ \mathbf{A}_{r|2}(i,j), & \text{if additive interaction}, \\ \mathbf{A}_{r|3}(i,j), & \text{if antagonistic interaction}. \end{cases} \end{align*}

To explicitly model the heterogeneity of interaction types, the heterogeneous graph $ \boldsymbol{\mathcal{G}}_{r} $ is decomposed into three type-specific homogeneous subgraphs. For the synergistic subgraph


\begin{align*} & \boldsymbol{\mathcal{G}}_{r,1} = (\mathcal{V}, \mathcal{E}_{r,1}, \mathbf{X}, \mathbf{A}_{r|1}), \end{align*}

the adjacency matrix $ \mathbf{A}_{r|1} \in \{0,1\}^{n \times n} $ satisfies $ \mathbf{A}_{r|1}(i,j) = 1 $ if $ (i,j) \in \mathcal{E}_{r,1} $, and 0 otherwise.

Similarly, the additive subgraph is defined as


\begin{align*} & \boldsymbol{\mathcal{G}}_{r,2} = (\mathcal{V}, \mathcal{E}_{r,2}, \mathbf{X}, \mathbf{A}_{r|2}), \end{align*}

where $ \mathbf{A}_{r|2}(i,j) = 1 $ if $ (i,j) \in \mathcal{E}_{r,2} $, and 0 otherwise.

The antagonistic subgraph is given by


\begin{align*} & \boldsymbol{\mathcal{G}}_{r,3} = (\mathcal{V}, \mathcal{E}_{r,3}, \mathbf{X}, \mathbf{A}_{r|3}), \end{align*}

where $ \mathbf{A}_{r|3}(i,j) = 1 $ if $ (i,j) \in \mathcal{E}_{r,3} $, and 0 otherwise.

Each subgraph adjacency matrix $ \mathbf{A}_{r|t} $ for $ t \in \{1,2,3\} $ is symmetric, reflecting the undirected nature of DDIs.

### Global drug interactive features by Tucker tensor decomposition

To effectively analyze and compress high-dimensional tensor data, we employ Tucker decomposition [23], a widely used tensor factorization technique that decomposes a high-order tensor into a lower order core tensor and a set of mode-specific factor matrices. This approach enables dimensionality reduction while preserving the essential structure of the data.

To extract global drug interaction features, we construct a third-order tensor $ \boldsymbol{\mathcal{A}}_{r} \in \mathbb{R}^{n \times n \times 3} $, where each frontal slice $ \boldsymbol{\mathcal{A}}_{r}(:,:, l) = \mathbf{A}_{r|l} $ corresponds to one type of drug interaction. The Tucker decomposition of $ \boldsymbol{\mathcal{A}}_{r} $ is given by


(1) \begin{align*}& \boldsymbol{\mathcal{A}}_{r} \approx \boldsymbol{G}_{r} \times_{1} \mathbf{U}_{r} \times_{2} \mathbf{V}_{r} \times_{3} \mathbf{J}_{r},\end{align*}

where $ \boldsymbol{G}_{r} \in \mathbb{R}^{R_{1} \times R_{2} \times R_{3}} $ is the core tensor, and $ \mathbf{U}_{r} \in \mathbb{R}^{n \times R_{1}} $, $ \mathbf{V}_{r} \in \mathbb{R}^{n \times R_{2}} $, $ \mathbf{J}_{r} \in \mathbb{R}^{3 \times R_{3}} $ are the mode-specific factor matrices. Here, $ R_{1}, R_{2}, R_{3} $ denote the ranks along each mode, and $ \times _{n} $ denotes the mode-$ n $ tensor–matrix product.

More formally, for a tensor $ \boldsymbol{\mathcal{T}} \in \mathbb{R}^{I_{1} \times I_{2} \times \cdots \times I_{N}} $ and a matrix $ \mathbf{M} \in \mathbb{R}^{J \times I_{n}} $, the mode-$ n $ product is defined as


(2) \begin{align*}& \boldsymbol{\mathcal{Y}} = \boldsymbol{\mathcal{T}} \times_{n} \mathbf{M} \in \mathbb{R}^{I_{1} \times \cdots \times I_{n-1} \times J \times I_{n+1} \times \cdots \times I_{N}},\end{align*}

with element-wise computation:


(3) \begin{align*}& \begin{aligned} \boldsymbol{\mathcal{Y}}&(i_{1}, \ldots, i_{n-1}, j, i_{n+1}, \ldots, i_{N}) \\ &= \sum_{i_{n}=1}^{I_{n}} \boldsymbol{\mathcal{T}}(i_{1}, \ldots, i_{n}, \ldots, i_{N}) \cdot \mathbf{M}(j, i_{n}). \end{aligned}\end{align*}

Expanding Equation (1) into its scalar form:


(4) \begin{align*}& \begin{aligned} \boldsymbol{\mathcal{A}}_{r}(i,j,k) \approx &\sum_{r_{1}=1}^{R_{1}} \sum_{r_{2}=1}^{R_{2}} \sum_{r_{3}=1}^{R_{3}} \big(\boldsymbol{G}_{r}(r_{1},r_{2},r_{3}) \\ &\cdot \mathbf{U}_{r}(i,r_{1}) \cdot \mathbf{V}_{r}(j,r_{2}) \cdot \mathbf{J}_{r}(k,r_{3})\big), \end{aligned}\end{align*}

indicating that each tensor element is approximated by a weighted linear combination of core tensor elements and factor matrix components.

We apply Tucker decomposition to the three-mode tensor $ \boldsymbol{\mathcal{A}}_{r} $ to extract global drug interaction features. As a higher order generalization of matrix singular value decomposition (SVD), Tucker decomposition uncovers low-rank structures that span all tensor modes. By factorizing $ \boldsymbol{\mathcal{A}}_{r} $ into a compact core and mode-specific factors, it captures latent global patterns and higher order dependencies across the synergistic, additive, and antagonistic dimensions. This decomposition aggregates information from the entire dataset, enabling a holistic understanding of DDIs beyond local feature patterns.

In contrast to methods that focus on localized regions of the data space, Tucker decomposition models comprehensive, multi-way correlations that may not be apparent from any single mode. In cell line-specific heterogeneous graphs, this framework enables effective modeling of complex drug combinations.

The mode matrix $ \mathbf{U}_{r} $, corresponding to the first mode of the decomposition, encodes the low-dimensional representations of drugs acting as interaction initiators. These embeddings capture structural and semantic regularities in a compressed latent space, supporting the modeling of diverse relational types. By leveraging $ \mathbf{U}_{r} $ as the global drug feature, we preserve rich inter-drug dependencies while maintaining efficiency and interpretability.

Our proposed framework establishes a hierarchical architecture for synergistic effect prediction, integrating pharmacological knowledge from both global (tensor-based) and local (graph-based) perspectives. Specifically, the global tensor-based representation provides a comprehensive structural characterization of drug interactions, which then guides the incorporation of local relational context through graph modeling to achieve robust and interpretable prediction.

### Local drug interactive feature by GTNs

In heterogeneous graphs, meta-paths serve as an essential mechanism for capturing higher order semantics by connecting node types through specific edge-type sequences. Formally, a meta-path $ p $ [24] is defined as a sequence of relations in graph $ \mathcal{G} $, expressed as


\begin{align*} & v_{1} \xrightarrow{t_{1}} v_{2} \xrightarrow{t_{2}} \cdots \xrightarrow{t_{l}} v_{l+1}, \end{align*}

where each $ t_{l} $ denotes the type of the $ l $th edge. The composite relation induced by $ p $ is denoted by $ R = t_{1} \circ t_{2} \circ \cdots \circ t_{l} $, linking the source $ v_{1} $ to the target $ v_{l+1} $. Although effective, meta-path construction traditionally relies on domain expertise and manual design, which may be suboptimal or incomplete.

To overcome this limitation, we adopt a GTN [25] that automatically learns meta-paths by jointly optimizing over heterogeneous adjacency structures. For each cell line $ r $, we stack three adjacency matrices $ \mathbf{A}_{r|1}, \mathbf{A}_{r|2}, \mathbf{A}_{r|3} $, corresponding to three drug interaction types, into a relationship tensor $ \boldsymbol{\mathcal{A}}_{r} \in \mathbb{R}^{n \times n \times 3} $.

At the $ l $th GTN layer, we apply convolution over the edge-type dimension using learnable parameters $ \mathbf{W}_{r}^{(l)} \in \mathbb{R}^{3 \times C} $, where $ C $ denotes the number of channels. A softmax over edge types yields weights used to compute soft aggregated adjacency maps:


(5) \begin{align*} \boldsymbol{\mathcal{Q}}_{r}^{(l)} &= \phi_{r}^{(l)}\left(\boldsymbol{\mathcal{A}}_{r}; \mathrm{softmax}(\mathbf{W}_{r}^{(l)})\right), \quad l = 1,\dots, L+1,\end{align*}

where $ \boldsymbol{\mathcal{Q}}_{r}^{(l)} \in \mathbb{R}^{n \times n \times C} $, and $ \phi _{r}^{(l)} $ denotes a $ 1 \times 1 $ convolutional operation.

The composite adjacency matrices are recursively constructed as


(6) \begin{align*} \boldsymbol{\mathcal{A}}_{r}^{(1)} &= \big\|_{c=1}^{C} {(\mathbf{D}_{c}^{(1)})}^{-1} \mathbf{Q}_{c}^{(1)} \mathbf{Q}_{c}^{(2)}, \notag\\ \boldsymbol{\mathcal{A}}_{r}^{(2)} &= \big\|_{c=1}^{C} {(\mathbf{D}_{c}^{(2)})}^{-1} \mathbf{A}_{c}^{(1)} \mathbf{Q}_{c}^{(3)}, \notag\\ &\cdots \notag\\ \boldsymbol{\mathcal{A}}_{r}^{(L)} &= \big\|_{c=1}^{C} {(\mathbf{D}_{c}^{(L)})}^{-1} \mathbf{A}_{c}^{(L-1)} \mathbf{Q}_{c}^{(L+1)},\end{align*}

where $ \big \| $ denotes concatenation across channels, $ \mathbf{Q}_{c}^{(l)} $ is the $ c $th channel of $ \boldsymbol{\mathcal{Q}}_{r}^{(l)} $, and $\mathbf{A}_{c}^{(l)} \in \mathbb{R}^{n\times n}$ is the $c$th channel of $\boldsymbol{\mathcal{A}}_{r}^{(l)} \in \mathbb{R}^{n\times n \times C}$. $ \mathbf{D}_{c}^{(l)} $ is the degree normalization matrix.

Graph convolution is then applied to the learned adjacency tensors:


(7) \begin{align*}& \boldsymbol{\mathcal{Z}}_{r} = \big\|_{c} \sigma\left(\tilde{\mathbf{D}}_{c}^{-1} \tilde{\mathbf{A}}_{c}^{(L)} \mathbf{X} \mathbf{W}_{r} \right), \quad \boldsymbol{\mathcal{Z}}_{r} \in \mathbb{R}^{n \times d \times C},\end{align*}

where $ \mathbf{X} \in \mathbb{R}^{n \times m} $ is the drug fingerprint matrix, which is obtained by a pretrained Deep Graph Infomax (DGI) model [26]. $\tilde{\mathbf{D}}_{c}$ is the degree matrix of $\tilde{\mathbf{A}}_{c}^{(L)}$. $ \mathbf{W}_{r} \in \mathbb{R}^{m \times d} $ is a trainable projection matrix, and $ \tilde{\mathbf{A}}_{c}^{(L)} $ adds self-loops on $ \mathbf{A}_{c}^{(L)} $.

For channel-level aggregation, we apply attention-based pooling:


(8) \begin{align*}& \mathbf{a}_{r} = \big\|_{c} \left( \frac{1}{n} \sum_{i=1}^{n} \mathbf{q}^\top \tanh\left( \mathbf{Z}_{r|c|i} \mathbf{P}_{r} + \mathbf{b}_{r} \right) \right) \in \mathbb{R}^{C},\end{align*}

where $ \mathbf{Z}_{r|c} \in \mathbb{R}^{n \times d} $ is the $ c $th channel of $ \boldsymbol{\mathcal{Z}}_{r} $, $ \mathbf{Z}_{r|c|i} $ denotes the $ i $th row, $ \mathbf{q} \in \mathbb{R}^{h} $ is the attention vector, and $ \mathbf{P}_{r} \in \mathbb{R}^{d \times h}, \mathbf{b}_{r} \in \mathbb{R}^{h} $ are learnable parameters.

The final local representation $ \mathbf{Z}_{r} \in \mathbb{R}^{n \times d} $ is computed as a weighted sum over channels:


(9) \begin{align*}& \mathbf{Z}_{r} = \sum_{c=1}^{C} \beta_{c} \mathbf{Z}_{r|c}, \quad \mathrm{where} \quad \beta_{c} = \frac{\exp(\mathbf{a}_{r}(c))}{\sum_{j} \exp(\mathbf{a}_{r}(j))}.\end{align*}

This representation integrates node embeddings from $ C $ distinct meta-path-based views, each with length up to $ L+1 $.

For each cell line $ r $, we integrate the Tucker-decomposed global feature $ \mathbf{U}_{r} $ and the graph-derived local feature $ \mathbf{Z}_{r} $ via concatenation


(10) \begin{align*}& \mathbf{H}_{r} = [\mathbf{U}_{r}; \mathbf{Z}_{r}],\end{align*}

where the $ i $th row of $\mathbf{H}_{r}$ denotes the interaction feature of drug $ i $ in cell line $ r $. This drug interactive feature is denoted as $\mathbf{h}_{r,i}$ in the following. This dual-source representation preserves both the intrinsic pharmacological properties of each drug and its context-specific behavioral profile shaped by the cellular microenvironment.

### Synergy score prediction

To more accurately predict the synergistic effect scores of drug combinations $(i, j)$ in a given cell line $r$, we integrate multiple sources of information:

- The cell line profile represented by its gene expression vector $\mathbf{g}_{r}$.
- Drug structural features $(\mathbf{s}_{i}, \mathbf{s}_{j})$, which is obtained by processing its atomic graph with a GCN. Starting from one-hot encoded atom features $H_{i}^{(0)}$, we apply $L$ graph convolution layers following the update rule ${H_{i}}^{(l+1)} = ReLU \big ( \tilde{D}_{i}^{-\frac{1}{2}}\tilde{A}_{i} \tilde{D}_{i}^{-\frac{1}{2}}{H_{i}}^{(l)} {W_{i}}^{(l)} \big )$. The final molecular representation $\mathbf{s}_{i} = \text{Global Average Pooling}(H_{i}^{(L)})$.
- Physicochemical fingerprints $(\mathbf{x}_{i}, \mathbf{x}_{j})$, corresponding to the $i$th and $j$th rows of the fingerprint matrix $\mathbf{X}$.
- Drug interactive features $(\mathbf{h}_{r,i}, \mathbf{h}_{r,j})$, obtained from the $i$th and $j$th rows of the global–local representation matrix $\mathbf{H}_{r}$.

Each of these four modalities is independently mapped into a unified latent space through four two-layer multilayer perceptrons (MLPs) with batch normalization:


(11) \begin{align*} \mathbf{g}_{r}^{\prime} &= \mathrm{MLP}_{g}(\mathbf{g}_{r}), \end{align*}

(12) \begin{align*} \mathbf{s}_{i}^{\prime} &= \mathrm{MLP}_{s}(\mathbf{s}_{i}), \quad \mathbf{s}_{j}^{\prime} = \mathrm{MLP}_{s}(\mathbf{s}_{j}), \end{align*}

(13) \begin{align*} \mathbf{x}_{i}^{\prime} &= \mathrm{MLP}_{x}(\mathbf{x}_{i}), \quad \mathbf{x}_{j}^{\prime} = \mathrm{MLP}_{x}(\mathbf{x}_{j}), \end{align*}

(14) \begin{align*} \mathbf{h}_{r,i}^{\prime} &= \mathrm{MLP}_{h}(\mathbf{h}_{r,i}), \quad \mathbf{h}_{r,j}^{\prime} = \mathrm{MLP}_{h}(\mathbf{h}_{r,j}). \end{align*}

The resulting features are concatenated to form a comprehensive vector:


(15) \begin{align*} & \mathbf{z}_{i,j,r} = \mathrm{concat}(\mathbf{g}_{r}^{\prime}, \mathbf{s}_{i}^{\prime}, \mathbf{s}_{j}^{\prime}, \mathbf{x}_{i}^{\prime}, \mathbf{x}_{j}^{\prime}, \mathbf{h}_{r,i}^{\prime}, \mathbf{h}_{r,j}^{\prime}).\end{align*}

Finally, the prediction is performed using a three-layer MLP with batch normalization:


(16) \begin{align*}& \hat{y}_{i,j,r} = \mathrm{MLP}_{\mathrm{pred}}(\mathbf{z}_{i,j,r}),\end{align*}

and the model is optimized with the mean squared error (MSE) loss:


(17) \begin{align*}& \mathcal{L}_{\mathrm{MSE}} = \frac{1}{|\mathcal{D}|} \sum_{(i,j,r) \in \mathcal{D}} \left( y_{i,j,r} - \hat{y}_{i,j,r} \right)^{2},\end{align*}

where $ y_{r,ij} $ is the ground truth synergy score and $\mathcal{D}$ is the training dataset.

### Complexity analysis

For each cell line $r$, the computational complexity comprises two components:

- Global drug interactive features: Tucker decomposition of the third-order tensor $\boldsymbol{\mathcal{A}}_{r} \in \mathbb{R}^{n\times n\times 3}$ requires computing the mode-1 factor matrix $\mathbf{U}_{r}$ via truncated SVD on the mode-1 unfolding, yielding a complexity of $O(n^{2}R_{1})$ [27].
- Local drug interactive features: The GTN operations consist of (i) softmax attention weight computation in Equation (5) with $O(C)$ complexity, and convolutional operation $\phi _{r}^{(l)}$ requiring $O(n^{2}C)$ operations; (ii) recursive composite adjacency matrix construction in Equation (6), which dominates the cost at $O(n^{3}CL)$; and (iii) graph convolution, attention pooling, and weighted aggregation in Equations 7,9 with combined complexity $O(n^{2}Cd + nmd + nCdh)$. The total complexity for this module is $O(n^{3}CL + n^{2}Cd + nmd + nCdh)$.

Aggregating across $R$ cell lines, the overall complexity becomes $O(Rn^{2} R_{1}+R(n^{3}CL + n^{2}Cd + nmd + nCdh))$. Since hyperparameters $m, d, h, L, C, R_{1}$ are fixed constants independent of data size, this simplifies to $O(n^{3}R)$. Notably, the complexity scales primarily with the number of drug combinations ($\approx n^{2}R$) rather than $n$ or $R$ individually. Consequently, for a fixed number of combinations, datasets with larger $n$ (more drugs, fewer cell lines) require greater computational time than those with smaller $n$ (fewer drugs, more cell lines).

## Experiments

This section presents the experimental protocol used to evaluate the performance of the proposed method. We begin by introducing the dataset and preprocessing procedures, followed by a formal definition of the drug synergy prediction task in the context of heterogeneous graphs. Subsequently, we describe the baseline methods for comparison, specify evaluation metrics aligned with clinical interpretability, and outline key hyperparameter settings for reproducibility.

### Drug combination datasets and synergy scoring

We evaluate the synergistic effects of drug combinations using three widely adopted benchmark datasets: O’Neil [28], ALMANAC [28], and CLOUD [28], as summarized in Table 2. These datasets provide extensive profiles of DDIs across a variety of cell lines. Each sample is represented as a triplet $(i, j, r)$, where $i$ and $j$ are two drugs, and $r$ denotes a specific cell line.

**Table 2:** Summary of drug combination synergy data, detailing dataset composition and interaction types scored by four metrics.

Category	Measure	O’Neil	CLOUD	ALMANAC
Dataset	Drugs	38	242	82
	Cell Lines	39	1	59
	Combinations	22 737	29 278	154 596
Loewe	Synergistic	1973	1430	60
	Additive	12 087	10 688	31 962
	Antagonistic	8677	17 160	122 574
Bliss	Synergistic	7829	5308	31 470
	Additive	10 484	3837	93 585
	Antagonistic	4424	20 133	29 541
ZIP	Synergistic	8806	6058	29 932
	Additive	10 803	3500	90 334
	Antagonistic	10 803	3500	90 334
HSA	Synergistic	11 958	9078	21 686
	Additive	8567	5211	91 749
	Antagonistic	2212	14 989	41 161

To quantify the degree of synergy, we utilize four well-established pharmacological metrics: Loewe additivity (Loewe), Bliss independence (Bliss), Zero interaction potency (ZIP), and Highest single agent (HSA) [29]. These scoring schemes provide continuous values, where higher scores generally indicate stronger synergistic effects.

For the purpose of classification, we adopt threshold values defined in prior studies [30][31] to categorize the effects of drug combinations into synergistic, additive, or antagonistic. The thresholds are defined as follows:

- Loewe: synergy if score $ \geq 30 $, additive if $ 0 < \mathrm{score} < 30 $, antagonism if $ \leq 0 $;
- Bliss: synergy if $ \geq 3.68 $, additive if $ -3.37 < \mathrm{score} < 3.68 $, antagonism if $ \leq -3.37 $;
- ZIP: synergy if $ \geq 3.87 $, additive if $ -3.37 < \mathrm{score} < 3.68 $, antagonism if $ \leq -3.37 $;
- HSA: synergy if $ \geq 2.64 $, additive if $ -4.48 < \mathrm{score} < 2.64 $, antagonism if $ \leq -4.48 $.

By applying these thresholds, we systematically label each drug–drug–cell line triplet into one of the three interaction types, facilitating downstream classification and regression tasks in synergy prediction.

### Data preprocessing and experimental setting

Building upon the classification of drug combination effects introduced in the previous section, we now describe the data preprocessing pipeline, which includes feature extraction for both drugs and cell lines. These features form the input to our drug synergy prediction framework.

**Drug representation.** Each drug is represented by two complementary types of features: molecular graph-derived features and chemical fingerprint embeddings.

We employ 300D Infomax fingerprints $X_{i}$ for each drug $i$, generated using a pretrained DGI model [26]. This unsupervised approach captures high-level structural and functional information of drug molecules, providing a compact yet informative representation for downstream tasks.

To facilitate fingerprint extraction, each drug is represented by its SMILES (Simplified Molecular Input Line Entry System) string, a widely adopted linear notation for molecular structures. Using RDKit [32], SMILES strings are parsed into molecular graphs, enabling efficient computation of atom-level features and connectivity. These graph-based representations are subsequently processed by a learnable GCN to extract structural features.

**Cell line representation.** For each cell line $r$, we use the gene expression profile $C_{r}$, sourced from the Cancer Cell Line Encyclopedia database (https://sites.broadinstitute.org/ccle/). These profiles provide genome-wide messenger RNA expression levels across a broad spectrum of cancer cell lines and serve as the primary modality to characterize cellular context in drug response modeling.

By extracting high-level chemical fingerprints for drugs and transcriptomic profiles for cell lines, our model integrates diverse sources of biological knowledge. These features provide a robust basis for learning drug–cell line interactions and predicting synergistic effects in combination therapy.

**Training configuration** Model training involves a series of carefully selected hyperparameters. The GTN module employs structural embeddings of dimension $ d = 32 $ to capture multi-scale drug interaction patterns. The number of channels is set to $ C = 2 $, providing a balance between representational capacity and overfitting risk. Optimization is performed using the Adam optimizer with an initial learning rate of $ 0.001 $.

To mitigate overfitting, we apply dropout regularization with a dataset-specific configuration. For the O’Neil and CLOUD datasets, which contain relatively limited samples, a dropout rate of $ 0.2 $ is used. In contrast, no dropout is applied to the large-scale ALMANAC dataset to maximize information retention.

The number of training epochs is set to 200 for the O’Neil and CLOUD datasets, reflecting their moderate complexity. For the ALMANAC dataset, which exhibits greater structural diversity and scale, the training is extended to 500 epochs to ensure sufficient convergence and capture of complex interaction patterns.

### Baseline models and evaluation metrics

To rigorously evaluate the performance of our proposed method, we formulate drug synergy prediction as both a regression and a classification task. In the regression setting, synergy scores are treated as continuous variables, consistent with the clinical objective of ranking drug combinations by their synergistic potential. We compare our model with three classical machine learning algorithms—Support Vector Regression (SVR) [33], Random Forest [34], and XGBoost [35]—widely adopted in biomedical research. Additionally, we benchmark against three recent state-of-the-art deep learning models: MatchMaker [17], PRODeepSyn [19], MGAE-DC [31], HypertranSynergy [36], and MFSynDCP [37], which are specifically designed for drug combination prediction. For a fair comparison, all baseline models and TensoGraph were trained using identical drug features, cell-line features, and drug SMILES, with feature preprocessing strictly following PRODeepSyn. All methods were evaluated under the same 10-fold cross-validation protocol.

To enable direct comparison with existing classification-based approaches, we further evaluate performance by discretizing synergy scores using predefined thresholds and categorizing drug combinations into three classes: synergistic (positive), additive, and antagonistic (negative). Additive and antagonistic combinations are grouped as the negative class, following conventions from prior studies.

For regression evaluation, we adopt four standard metrics: MSE, root mean squared error (RMSE), Pearson correlation coefficient (PCC), and 95% confidence interval (CI). Each model is assessed using 10-fold cross-validation, reporting the mean and standard deviation across folds. For classification performance, we employ multiple evaluation metrics to comprehensively assess both predictive accuracy and robustness, including Area Under the Receiver Operating Characteristic Curve (AUC), Area Under the Precision–Recall Curve (AUPR), Accuracy (ACC), Precision, Recall, and Cohen’s Kappa.

### Performance evaluation across all cell lines

We conduct a comprehensive evaluation of our proposed model for drug combination synergy prediction across diverse cell lines, employing a dual-task evaluation framework. This framework includes (i) regression of continuous synergy scores to capture the nuanced quantitative interactions between drugs, reflecting the complex pharmacodynamics, and (ii) binary classification to distinguish synergistic from non-synergistic drug pairs based on clinically relevant thresholds, facilitating real-world therapeutic decision-making. To ensure robustness and statistical reliability, all experiments are performed using a stratified 10-fold cross-validation scheme, which accounts for underlying heterogeneity in cell line-specific drug responses and mitigates potential biases from sample imbalance.

**Figure 2:**
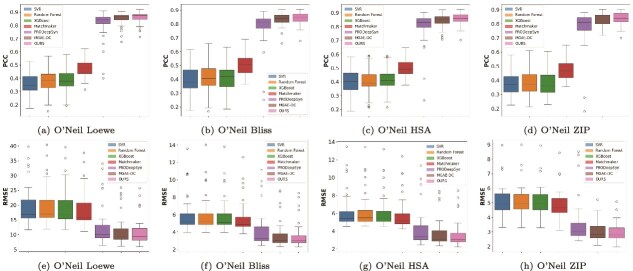
Comparative performance of drug synergy prediction models across all cell lines on the O’Neil dataset. This figure presents the comparative performance of seven drug synergy prediction methods, including SVR, Random Forest, XGBoost, Matchmaker, PRODeepSyn, MGAE-DC, and TensoGraph (OURS), evaluated across all cell lines in the O’Neil dataset. Figures (a)–(d) report the PCCs under four synergy scoring schemes: (a) Loewe, (b) Bliss, (c) HSA, and (d) ZIP, reflecting the correlation between predicted and experimental synergy values. Figures (e)–(h) present the corresponding RMSE values of the predicted synergy scores under the same four scoring schemes: (e) Loewe, (f) Bliss, (g) HSA, and (h) ZIP, indicating the overall predictive accuracy of each method.

Figure 2 presents the regression results on the O’Neil dataset, a benchmark containing well-characterized drug combinations with diverse mechanisms of action and varying chemical properties. Our model consistently outperforms the strongest baseline, MGAE-DC, across all four commonly used synergy metrics (Loewe, Bliss, HSA, ZIP). Specifically, it achieves the lowest RMSE values 12.27 for Loewe, 3.95 for Bliss and HSA, and 3.24 for ZIP, representing a 3.5%–6.2% improvement. The PCCs (ranging from 0.84 to 0.86) indicate a strong concordance between predicted and true synergy scores, highlighting the model’s ability to capture intricate drug interaction effects influenced by factors such as target pathway crosstalk, drug dosage dependencies, and off-target activities. Furthermore, we observe reduced variance across folds, demonstrating the model’s stability and generalizability across moderately sized datasets that include chemically and biologically diverse compounds. Figure 2 also visualizes the distribution of RMSE and PCC scores across individual cell lines, where our model consistently shows lower prediction errors and higher correlation coefficients compared with competing methods. This robustness across multiple synergy quantification metrics indicates that our approach effectively integrates both global drug features and cell-specific contextual information, enabling reliable predictions even for cell lines with heterogeneous drug sensitivity profiles or complex resistance mechanisms.

**Figure 3:**
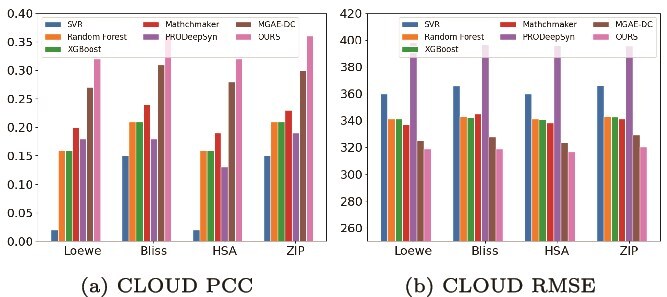
Performance evaluation across all cell lines on the CLOUD dataset. This figure summarizes the performance of seven computational models on the CLOUD dataset encompassing a diverse set of drug combinations and cell lines. Figure (a) shows the PCCs of predicted versus observed synergy scores, while figure (b) presents the corresponding RMSE values. Each bar denotes the mean performance for a given synergy scoring model (Loewe, Bliss, HSA, or ZIP), averaged across all drug–cell line combinations.

We extend the evaluation to larger scale datasets, with Fig. 3 presenting results on the CLOUD dataset, which includes a broader range of drug classes and exhibits increased sample heterogeneity. Our method demonstrates consistent improvements, compared with the second best MGAE-DC, the Loewe RMSE decreases from 18.17 to 17.85, while PCC improves from 0.26 to 0.32. These results highlight the scalability and robustness of our model, emphasizing its ability to capture complex synergistic mechanisms such as synthetic lethality and compensatory pathway inhibition.

**Figure 4:**
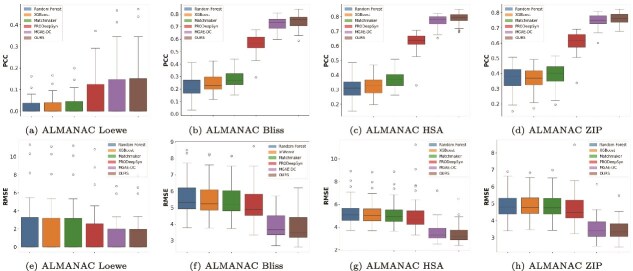
Performance evaluation across all cell lines on the ALMANAC dataset. This figure compares the performance of six drug synergy prediction models, including Random Forest, XGBoost, Matchmaker, PRODeepSyn, MGAE-DC, and TensoGraph (OURS), evaluated across all cell lines in the ALMANAC dataset. Figures (a)–(d) illustrate the PCCs of predicted and experimental synergy scores for four scoring models: (a) Loewe, (b) Bliss, (c) HSA, and (d) ZIP. Figures (e–h) show the corresponding RMSE values under the same four scoring schemes: (e) Loewe, (f) Bliss, (g) HSA, and (h) ZIP. The SVR method is excluded from the comparison because it failed to converge under computational constraints and data complexity.

On the large-scale ALMANAC dataset (Fig. 4), which includes extensive combinatorial screening data across a wide panel of cancer cell lines and drug modalities, our model achieves notable improvements despite the dataset’s increased complexity. Traditional models like SVR fail to converge due to the scale and heterogeneity of the data. Specifically, for the Loewe metric, RMSE reaches 10.75, with a PCC of 0.77, demonstrating the model’s ability to capture subtle interaction patterns influenced by mechanisms such as pathway redundancy and dynamic cellular rewiring. These results underscore the model’s potential for real-world large-scale drug discovery, where identifying effective drug combinations is crucial to overcoming drug resistance and improving the therapeutic index. The specific numerical results for these three datasets can be found in the section 1 of the Supplementary Materials (Tables S1–S3).

Although drug synergy prediction is traditionally framed as a regression problem, prior literature often treats it as a binary classification task to identify synergistic combinations with clinical relevance. To enable direct comparison, we convert predicted synergy scores into binary labels using clinically established thresholds. Table 3 shows the classification metrics on the O’Neil dataset using Loewe scores. Both our model and MGAE-DC achieve leading AUC and accuracy of 0.95. However, our model outperforms all competitors in AUPR (0.70), F1-score (0.80), and Cohen’s Kappa (0.64), which are crucial metrics for evaluating performance under severe class imbalance and assessing agreement beyond random chance. This demonstrates the model’s strong discriminative ability in identifying synergistic drug pairs amidst a diverse set of chemical scaffolds and cellular contexts.

**Table 3:** Classification performance of different methods for predicting drug–drug synergistic effects on the O’Neil using Loewe scoring.

Method	AUC	AUPRC	ACC	F1	Kappa
SVR	0.69 $\pm $ 0.04	0.39 $\pm $ 0.02	0.91 $\pm $ 0.003	0.41 $\pm $ 0.11	0.06 $\pm $ 0.04
Random Forest	0.70 $\pm $ 0.03	0.28 $\pm $ 0.07	0.91 $\pm $ 0.005	0.39 $\pm $ 0.11	0.11 $\pm $ 0.05
XGBoost	0.71 $\pm $ 0.04	0.30 $\pm $ 0.08	0.91 $\pm $ 0.004	0.46 $\pm $ 0.13	0.06 $\pm $ 0.04
Matchmaker	0.71 $\pm $ 0.04	0.30 $\pm $ 0.08	0.91 $\pm $ 0.004	0.46 $\pm $ 0.13	0.06 $\pm $ 0.04
PRODeepSyn	0.93 $\pm $ 0.01	0.63 $\pm $ 0.04	0.94 $\pm $ 0.01	0.67 $\pm $ 0.04	0.57 $\pm $ 0.02
MGAE-DC	0.95 $\pm $ 0.01	0.68 $\pm $ 0.02	0.95 $\pm $ 0.01	0.78 $\pm $ 0.05	0.61 $\pm $ 0.02
MFSynDCP	0.93 $\pm $ 0.005	0.65$\pm $ 0.002	0.91 $\pm $ 0.006	0.78 $\pm $ 0.02	**0.70 $\pm $ 0.01**
HypertranSynergy	0.93 $\pm $ 0.01	0.69 $\pm $ 0.02	0.94 $\pm $ 0.01	0.66 $\pm $ 0.02	0.59$\pm $ 0.02
OURS	**0.95 $\pm $ 0.01**	**0.70 $\pm $ 0.02**	**0.95 $\pm $ 0.002**	**0.80 $\pm $ 0.04**	0.64 $\pm $ 0.02

Bold indicates the best result, and underlined indicates the second best result.

These results indicate that our model not only achieves top-tier prediction accuracy but also demonstrates consistent performance across different datasets, synergy metrics, and evaluation methods. By integrating both global and local drug interaction features specific to each cell line, the model captures essential biological and chemical factors that influence drug synergy. This holistic approach, considering both broad and context-specific information, leads to more accurate and reliable predictions, making our method a valuable tool for real-world drug synergy prediction.

### Performance evaluation on specific cell lines

**Figure 5:**
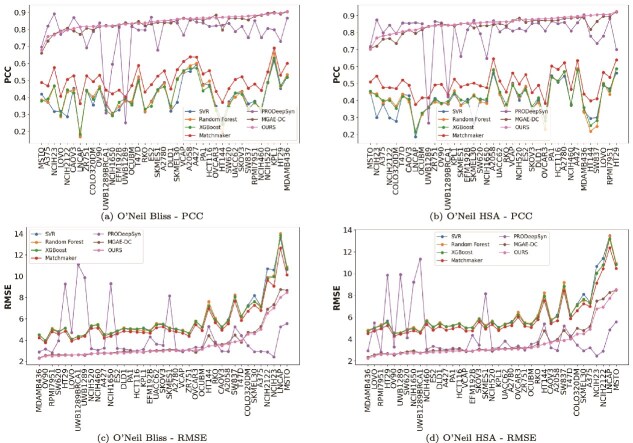
Performance evaluation on specific cell lines in the O’Neil dataset. Performance comparison of seven drug synergy prediction methods (SVR, Random Forest, XGBoost, Matchmaker, PRODeepSyn, MGAE-DC, and OURS) across 39 individual cell lines in the O’Neil dataset. Figures (a) and (b) display PCCs under Bliss and HSA scoring schemes, respectively, while figures (c) and (d) report RMSE values for the same settings. Each line corresponds to a model, and the horizontal axis enumerates distinct cell lines.

To systematically explore cell line-specific variations in drug synergy prediction, we conduct comprehensive performance assessments across individual cell lines in three major datasets: O’Neil, ALMANAC, and CLOUD. This analysis addresses the critical biological premise that drug combination efficacy is highly dependent on the heterogeneous genomic and molecular characteristics of different cellular contexts. For each dataset, we evaluate model performance using four widely adopted synergy scoring metrics—Loewe, Bliss, HSA, and ZIP—to ensure robust validation across different mathematical formulations of drug synergy. Representative results for the O’Neil dataset are presented in the main text (Fig. 5), while the full cross-dataset evaluations, including ALMANAC and CLOUD results under all four scoring metrics, are detailed in the Supplementary Materials (Figures S1–S5).

**Performance across O’Neil cell lines.**  Figure 5 presents the performance comparison of seven drug synergy prediction methods (SVR, Random Forest, XGBoost, Matchmaker, PRODeepSyn, MGAE-DC, and OURS) across 39 cell lines in the O’Neil dataset under Bliss independence and HSA scoring schemes. As illustrated in Fig. 5a and b, our model demonstrates robust and consistently superior PCC values across the majority of cell lines for both scoring methods. While traditional machine learning approaches (SVR, Random Forest, XGBoost) exhibit highly variable performance with PCC values frequently dropping below 0.4, our method maintains PCC values predominantly above 0.8, with peak performance reaching 0.92 in several cell lines including A2780 (ovarian carcinoma), NCIH2122 (non-small cell lung cancer), and MDAMB231 (triple-negative breast cancer). Notably, the performance exhibits greater stability across various cell lines compared with the second best-performing method, MGAE-DC. The root mean square error (RMSE) analysis presented in Figs 5c and d reveals similarly compelling results. Our method achieves the lowest RMSE values in 15 out of 39 cell lines for both Bliss and HSA scoring, with particularly notable performance in MDAMB436, KPL1, and A2780. Importantly, while baseline methods show substantial RMSE fluctuations across cell lines with some exceeding 14 for challenging cases, our approach maintains relatively stable error rates, demonstrating superior generalization capability across diverse cellular contexts. Analysis across all four synergy metrics reveals consistent trends (Supplementary Figure S1).

**Figure 6:**
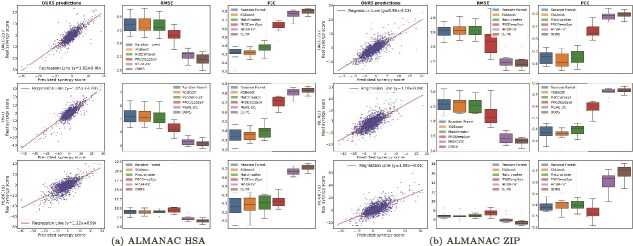
Comparative analysis on representative cell lines from the ALMANAC dataset. Evaluation of TensoGraph (OURS) and baseline methods across three representative cell lines in the ALMANAC dataset under (a) HSA and (b) ZIP scoring schemes. Each panel includes regression visualization between predicted and true synergy scores (left), RMSE comparison (middle), and PCC distribution (right).

**Detailed analysis of representative cell lines.** To further elucidate model behavior across distinct cellular contexts, we performed a detailed evaluation on representative cell lines from the ALMANAC dataset. Figure 6 summarizes the comparative performance of TensoGraph (OURS) and baseline methods under the HSA and ZIP synergy scoring schemes. Across all examined cell lines, TensoGraph exhibits a strong linear relationship between predicted and observed synergy scores, with regression lines closely following the ideal $y = x$ trend. The RMSE and PCC comparisons further demonstrate that TensoGraph consistently achieves lower prediction errors and higher correlation than competing models, confirming its robust generalization across both synergy formulations and heterogeneous cellular environments. These findings underscore the model’s ability to capture subtle drug–cell interaction patterns that traditional machine learning methods often overlook. Comprehensive results covering three benchmark datasets (ALMANAC, O’Neil, and CLOUD) under four scoring schemes (HSA, ZIP, Loewe, and Bliss) are provided in the Supplementary Materials (Figures S4–S6). These complementary analyses further corroborate the superior stability and predictive accuracy of TensoGraph across diverse datasets and scoring paradigms.

**Cell line-specific embedding analysis.** To assess whether the proposed model captures biologically meaningful, cell line-specific interaction patterns, we analyzed the learned embeddings of a representative compound, BEZ-235, across the 39 cell lines in the O’Neil dataset. The high-dimensional drug–cell interaction embeddings were projected into two dimensions using t-SNE for visualization. As shown in Figure S3 of the Supplementary Materials, cell lines exhibit clear clustering according to tissue of origin across six major cancer types. This result indicates that the learned representations encode tissue-specific pharmacogenomic characteristics, reflecting context-dependent drug–cell interactions rather than purely chemical or cell-intrinsic features.

### Ablation study

To rigorously assess the individual and synergistic contributions of the core architectural components in our proposed framework, we conduct a systematic ablation study through progressive module exclusion while maintaining the foundational network structure. This hierarchical ablation design enables precise quantification of three critical feature extraction mechanisms: (i) molecular graph encoding via GCNs (denoted as Component A), which captures topological and chemical properties of drug molecules; (ii) local relational feature extraction through GTNs, which models pairwise drug interactions and context-dependent synergistic patterns; and (iii) global multi-way interaction modeling via Tucker decomposition, which captures higher order relationships across the drug–drug–cell line tensor space. We systematically construct six model configurations following a carefully designed exclusion hierarchy to isolate the contribution of each component in Table 4. For detailed explanations of each setting, please refer to Section 4 of the Supplementary Materials.

**Table 4:** The results of the ablation study.

Method	MSE	RMSE	CI	PCC
OURS w/o A&Tucker&GTN	167.73 $\pm $ 13.11	12.94 $\pm $ 0.50	[156.22, 179.25]	0.82 $\pm $ 0.02
OURS w/o Tucker&GTN	163.28 $\pm $ 10.13	12.77 $\pm $ 0.39	[154.39, 172.17]	0.83 $\pm $ 0.02
OURS w/o A&GTN	157.43 $\pm $ 13.55	12.54 $\pm $ 0.54	[145.54, 169.32]	**0.84 $\pm $ 0.02**
OURS w/o A&Tucker	154.31 $\pm $ 11.02	12.41 $\pm $ 0.44	[144.64, 163.99]	**0.84 $\pm $ 0.01**
OURS w/o A	154.69 $\pm $ 10.45	12.43 $\pm $ 0.42	[145.51, 163.87]	**0.84 $\pm $ 0.01**
OURS	**150.67 $\pm $ 10.07**	**12.27 $\pm $ 0.41**	**[141.83, 159.51]**	**0.84 $\pm $ 0.02**

Bold indicates the best result, and underlined indicates the second best result.

Table 4 presents comprehensive performance metrics across all ablation variants, evaluated on the O’Neil dataset using Bliss independence scoring. The TensoGraph (OURS) achieves optimal performance, while the minimal baseline (OURS w/o A&Tucker&GTN) exhibits the poorest results, representing an 11.3% performance degradation. Analysis of specific ablation comparisons elucidates individual component contributions. Comparing OURS with OURS w/o A reveals that GCN-based molecular graph encoding contributes a 4.02 MSE reduction. When interaction modeling components are absent, comparing OURS w/o Tucker&GTN with OURS w/o A&Tucker&GTN shows a 4.45 reduction attributable to GCN, underscoring the importance of explicit molecular topology encoding beyond fixed fingerprints. GTN’s contribution is substantial, yielding a 13.42 MSE reduction when comparing OURS w/o A&Tucker with OURS w/o A&Tucker&GTN. The GTN’s ability to model context-dependent pairwise drug interactions through attention mechanisms captures synergistic patterns emerging from specific combination contexts. Similarly, Tucker decomposition contributes a 10.30 MSE reduction, as evidenced by comparing OURS w/o A&GTN with OURS w/o A&Tucker&GTN. Tucker decomposition captures higher order interactions across the drug–drug–cell line tensor space, modeling complex multi-way relationships that complement pairwise interactions. The comparable improvement magnitudes from GTN and Tucker suggest these components capture complementary aspects of drug synergy—local pairwise interactions versus global tensor patterns—with their combination yielding synergistic benefits that establish the necessity of the comprehensive multicomponent architecture.

**Figure 7:**
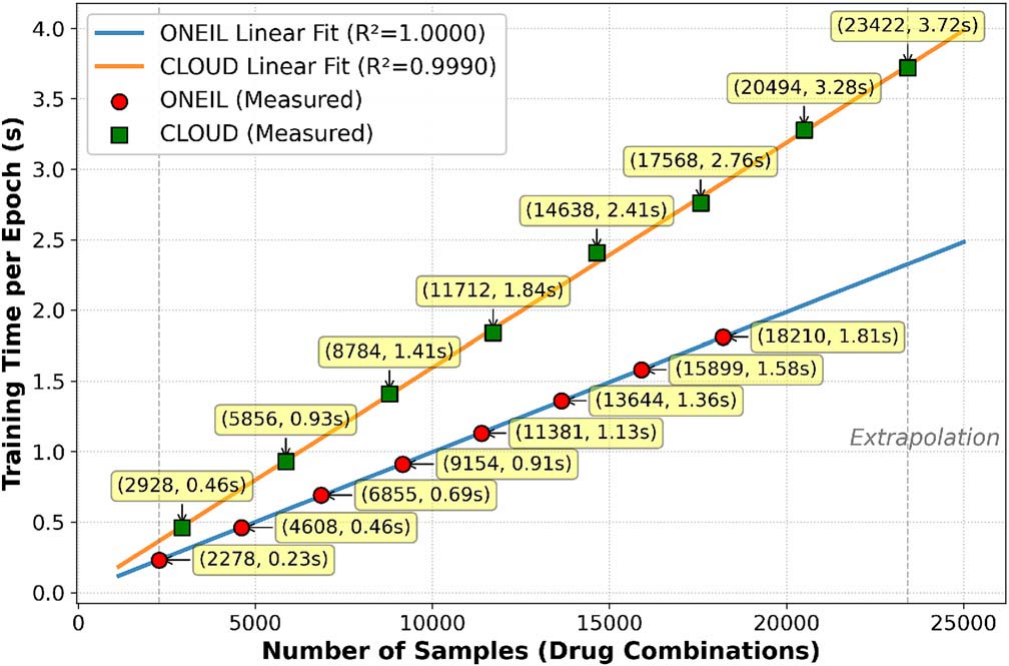
The influence of the number of drug combinations on training time.

Beyond architectural contribution analysis, we further examine the computational behavior of the proposed framework under controlled settings. By fixing all model components and hyperparameters and varying only the training set size, we analyze how the per-epoch training cost scales with the number of drug combinations. Figure 7 illustrates the relationship between per-epoch training time and the number of drug combinations on both O’Neil and CLOUD datasets. Each dataset is evaluated across eight configurations with varying training set sizes, ranging from 2278 to 18 210 combinations for O’Neil and from 2928 to 23 422 combinations for CLOUD. The linear fits demonstrate that for a fixed dataset (i.e. fixed $n$ and $R$), training time scales linearly with the number of training samples, confirming predictable computational costs. More importantly, comparing the two datasets reveals the impact of dataset characteristics on computational efficiency. Although both datasets contain comparable total numbers of combinations, CLOUD exhibits a significantly steeper slope due to its larger $n$ (more drugs, fewer cell lines). This empirical observation directly validates our theoretical complexity analysis of $O(n^{3}R)$ in *Complexity analysis*: for a fixed number of combinations, computational time increases with $n$, as datasets with more drugs require greater computational effort than those with more cell lines.

### Parameter sensitivity analysis

Model hyperparameters critically influence the trade-off between expressiveness and generalizability in drug synergy prediction. We conducted a grid search on the O’Neil–Loewe dataset over three key parameters: the GTN embedding dimension $d$, the number of aggregation channels $C$, and the downstream MLP embedding size, to quantify their influence on performance.

As shown in Supplementary Materials Fig. S6, performance is sensitive to $d$, with optimal accuracy achieved at $d=32$, balancing representational capacity and computational efficiency. The model remains robust to moderate variations around this value. Increasing $C$ yields diminishing returns, with marginal performance gains at substantially higher computational cost, leading us to select $C=2$. Performance improves with MLP embedding size until saturating at 8192; larger embeddings result in overparameterization. We therefore adopt an embedding size of 8192 to ensure stable and high-fidelity predictions.

## Conclusion

We present TensoGraph, a deep learning framework integrating molecular graph learning, heterogeneous graph modeling, and tensor decomposition for drug combination synergy prediction. Starting from drug SMILES representations, molecular graphs are encoded via GCNs to capture key structural and chemical features. Cell line-specific heterogeneous graphs are then constructed to represent drug–drug and drug–cell relationships. By unifying local relational features extracted by graph transformers with global interaction patterns learned through Tucker decomposition, TensoGraph effectively models both fine-grained and system-level determinants of drug efficacy.

Across multiple benchmark datasets, TensoGraph consistently surpasses state-of-the-art baselines, with notable gains on the large and heterogeneous ALMANAC dataset. The model also learns biologically meaningful representations, as evidenced by the clustering of cell lines according to tissue origin, underscoring its interpretability and potential for pharmacogenomic insight.

While the proposed framework demonstrates strong empirical performance, several aspects warrant further exploration. In particular, the selection of tensor ranks in Tucker decomposition introduces additional modeling choices, and adaptive rank selection may further enhance flexibility and robustness. Moreover, extending the framework to more challenging scenarios involving unseen drugs or cell lines remains an important direction for future work.

Future extensions may incorporate multi-omics data (e.g. gene expression, mutation, or epigenetic profiles) to refine cell state representation, and dose–response modeling to capture dynamic therapeutic effects beyond single synergy scores. Applying TensoGraph to patient-derived or single-cell datasets can further enable individualized combination strategies for precision oncology. Collectively, these results establish TensoGraph as a robust, interpretable, and extensible framework for data-driven drug synergy discovery and translational decision support.

**Key Points**

- We propose TensoGraph, a novel tensor-transformer framework that integrates Tucker decomposition and graph transformer networks to capture both global and local drug interaction patterns for synergistic combination prediction.
- We construct cell line-specific heterogeneous graphs with multi-relational edges, modeling context-dependent drug–drug interactions explicitly.
- Extensive experiments on multiple benchmark datasets demonstrate that TensoGraph consistently outperforms state-of-the-art baselines in both regression and classification tasks, improving prediction accuracy and biological interpretability.

## Supplementary Material

supplementary_bbag157

## Data Availability

The datasets used in this study are all publicly available. The source code for TensoGraph can be downloaded from GitHub (https://github.com/LiminLi-xjtu/TensoGraph).

## References

[1] Csermely P, Korcsmáros T, Kiss HJM et al. Structure and dynamics of molecular networks: a novel paradigm of drug discovery: a comprehensive review. *Pharmacol Ther* 2013;138:333–408. 10.1016/j.pharmthera.2013.01.016 23384594 PMC3647006

[2] Chou TC . Drug combination studies and their synergy quantification using the Chou-Talalay method. *Cancer Res* 2010;70:440–6. 10.1158/0008-5472.CAN-09-1947 20068163

[3] Yejin K, Zheng S, Tang J et al. Anticancer drug synergy prediction in understudied tissues using transfer learning. *J Am Med Inform Assoc* 2021;28:42–51. 10.1093/jamia/ocaa212 33040150 PMC7810460

[4] Vitiello PP, Martini G, Mele L et al. Vulnerability to low-dose combination of irinotecan and niraparib in ATM-mutated colorectal cancer. *J Exp Clin Cancer Res* 2021;40:15. 10.1186/s13046-020-01811-8 33407715 PMC7789007

[5] Giles TD, Weber MA, Basile J et al. Efficacy and safety of nebivolol and valsartan as fixed-dose combination in hypertension: a randomised, multicentre study. *Lancet* 2014;383:1889–98. 10.1016/S0140-6736(14)60614-0 24881993

[6] Sun X, Vilar S, Tatonetti NP. High-throughput methods for combinatorial drug discovery. *Sci Transl Med* 2013;5:205. 10.1126/scitranslmed.3006667 24089409

[7] Liu J, Gefen O, Ronin I et al. Effect of tolerance on the evolution of antibiotic resistance under drug combinations. *Science* 2020;367:200–4. 10.1126/science.aay3041 31919223

[8] Zheng W, Sun W, Simeonov A. Drug repurposing screens and synergistic drug-combinations for infectious diseases. *Br J Pharmacol* 2018;175:181–91. 10.1111/bph.13895 28685814 PMC5758396

[9] Azam F, Vazquez A. Trends in phase ii trials for cancer therapies. *Cancers* 2021;13:178. 10.3390/cancers13020178 33430223 PMC7825663

[10] Rikkala PR, Jha S, Pore D et al. A review on drug combination strategy for pharma life cycle management. *J Biol Today’s World* 2020;9:215.

[11] Jiménez-Luna J, Grisoni F, Schneider G. Drug discovery with explainable artificial intelligence. *Nat Mach Intell* 2020;2:573–84. 10.1038/s42256-020-00236-4

[12] Li X, Xu Y, Cui H et al. Prediction of synergistic anti-cancer drug combinations based on drug target network and drug induced gene expression profiles. *Artif Intell Med* 2017;83:35–43. 10.1016/j.artmed.2017.05.008 28583437

[13] Yen S, Daugherty AC, Schroeder EA et al. Synergistic drug combinations from electronic health records and gene expression. *J Am Med Inform Assoc* 2017;24:565–76. 10.1093/jamia/ocw161 27940607 PMC6080645

[14] Celebi R, Iv OBDW, Movva R et al. In-silico prediction of synergistic anti-cancer drug combinations using multi-omics data. *Sci Rep* 2019;9:8949. 10.1038/s41598-019-45236-6 31222109 PMC6586895

[15] Jeon M, Kim S, Park S et al. In silico drug combination discovery for personalized cancer therapy. *BMC Syst Biol* 2018;12:59–67. 10.1186/s12918-018-0546-1 29560824 PMC5861486

[16] Preuer K, Lewis RPI, Hochreiter S et al. DeepSynergy: predicting anti-cancer drug synergy with deep learning. *Bioinformatics* 2017;34:1538–46. 10.1093/bioinformatics/btx806 PMC5925774 29253077

[17] Kuru HI, Tastan O, Cicek E. MatchMaker: a deep learning framework for drug synergy prediction. *IEEE/ACM Trans Comput Biol Bioinform* 2021;19:2334–44. 10.1109/TCBB.2021.3086702 34086576

[18] Wang J, Liu X, Shen S et al. DeepDDS: deep graph neural network with attention mechanism to predict synergistic drug combinations. *Brief Bioinform* 2022;23:bbab390. 10.1093/bib/bbab390 34571537

[19] Xiaowen W, Zhu H, Jiang Y et al. PRODeepSyn: predicting anticancer synergistic drug combinations by embedding cell lines with protein–protein interaction network. *Brief Bioinform* 2022;23:bbab587. 10.1093/bib/bbab587 PMC8921631 35043159

[20] Liu Q, Xie L. TranSynergy: mechanism-driven interpretable deep neural network for the synergistic prediction and pathway deconvolution of drug combinations. *PLoS Comput Biol* 2021;17:e1008653–22. 10.1371/journal.pcbi.1008653 33577560 PMC7906476

[21] Balaevi I, Allen C, Hospedales TM. TuckER: tensor factorization for knowledge graph completion. Proceedings of the 2019 Conference on Empirical Methods in Natural Language Processing and the 9th International Joint Conference on Natural Language Processing (EMNLP-IJCNLP), pp. 5185–94. 2019.

[22] Saragadam V, Balestriero R, Veeraraghavan A et al. DeepTensor: low-rank tensor decomposition with deep network priors. *IEEE Trans Pattern Anal Mach Intell* 2024;46:10337–48. 10.1109/TPAMI.2024.3450575 39190515

[23] Tucker L . Some mathematical notes on three-mode factor analysis. *Psychometrika* 1966;31:279–311. 10.1007/BF02289464 5221127

[24] Wang X, Ji H, Shi C et al. Heterogeneous graph attention network. The World Wide Web Conference 2019;2022–32.

[25] Yun S, Jeong M, Kim R et al. Graph transformer networks. Advances in Neural Information Processing Systems 2019;32.

[26] Zagidullin B, Wang Z, Guan Y et al. Comparative analysis of molecular fingerprints in prediction of drug combination effects. *Brief Bioinform* 2021;22:bbab291. 10.1093/bib/bbab291 PMC8574997 34401895

[27] Ahmadi-Asl S, Abukhovich S, Asante-Mensah MG et al. Randomized algorithms for computation of Tucker decomposition and higher order SVD (HOSVD). *IEEE Access* 2021;9:28684–706. 10.1109/ACCESS.2021.3058103

[28] Bulat Z, Aldahdooh J, Zheng S et al. DrugComb: an integrative cancer drug combination data portal. *Nucleic Acids Res* 2019;47:W43–51. 10.1093/nar/gkz337 31066443 PMC6602441

[29] O’Neil J, Benita Y, Feldman I et al. An unbiased oncology compound screen to identify novel combination strategies. *Mol Cancer Ther* 2016;15:1155–62. 10.1158/1535-7163.MCT-15-0843 26983881

[30] Liu H, Zhang W, Zou B et al. DrugCombDB: a comprehensive database of drug combinations toward the discovery of combinatorial therapy. *Nucleic Acids Res* 2020;48:D871–81. 10.1093/nar/gkz1007 31665429 PMC7145671

[31] Zhang P, Tu S. MGAE-DC: predicting the synergistic effects of drug combinations through multi-channel graph autoencoders. *PLoS Comput Biol* 2023;19:e1010951. 10.1371/journal.pcbi.1010951 36867661 PMC10027223

[32] Bento AP, Hersey A, Félix E et al. An open source chemical structure curation pipeline using RDKit. *J Chem* 2020;12:1–16. 10.1186/s13321-020-00456-1 PMC7458899 33431044

[33] Drucker H, Burges CJ, Kaufman L et al. Support vector regression machines. *Adv Neural Inf Proces Syst* 1996;9:155–61.

[34] Breiman L . Random forests. *Mach Learn* 2001;45:5–32. 10.1023/A:1010933404324

[35] Chen T, Guestrin C. XGBoost: a scalable tree boosting system. Proceedings of the 22nd ACM SIGKDD international conference on knowledge discovery and data mining, pp. 785–94. New York, NY, USA: ACM, 2016.

[36] Wang W, Yuan G, Wan S et al. A granularity-level information fusion strategy on hypergraph transformer for predicting synergistic effects of anticancer drugs. *Brief Bioinform* 2024;25:bbad522. 10.1093/bib/bbad522 PMC10796255 38243692

[37] Dong Y, Chang Y, Wang Y et al. MFSynDCP: multi-source feature collaborative interactive learning for drug combination synergy prediction. *BMC Bioinformatics* 2024;25:140. 10.1186/s12859-024-05765-y 38561679 PMC10985899

