# Supplementary material for: TensoGraph: a tensor–transformer framework for global–local drug synergy prediction on heterogeneous graphs
Source: Brief Bioinform. 2026 Apr 14;27(2):bbag157. doi: 10.1093/bib/bbag157 (PMC13076947; doi:10.1093/bib/bbag157)
Supplement: supplementary_bbag157 [file supplementary_bbag157.pdf]

# Supplementary Materials

## TensoGraph: Tensor-Based Global–Local Modeling for Drug Synergy Prediction in Heterogeneous Graphs

# 1 Performance Evaluation Across All Cell Lines

## 1.1 Regression Results on O'Neil dataset

Table S1 presents comprehensive regression performance metrics on the O'Neil dataset across seven prediction methods evaluated under four synergy scoring schemes (Loewe, Bliss, HSA, and ZIP), providing detailed quantitative evidence supporting the performance trends visualized in Figure 2 in the main text. Our proposed method demonstrates consistent superiority across all evaluation metrics and synergy frameworks. Under Loewe additivity scoring, TensoGraph achieves MSE = 150.67 ± 10.07, RMSE = 12.27 ± 0.41, and PCC = 0.84 ± 0.02, with a 95% confidence interval of [141.83, 159.51], representing 11.44 improvement in MSE compared to the second-best method MGAE-DC (MSE = 162.11 ± 17.03). For Bliss independence, our method attains the lowest prediction error (MSE = 15.78 ± 3.23, RMSE = 3.95 ± 0.40) with the highest correlation (PCC = 0.85 ± 0.02), outperforming MGAE-DC by 7.4% in its MSE. Similarly, under HSA scoring, TensoGraph achieves MSE = 15.70 ± 2.08 and PCC = 0.85 ± 0.01, demonstrating a 10.7% MSE reduction of the second-best method MGAE-DC with notably tighter confidence intervals [13.88, 17.53] versus [15.79, 19.38], indicating enhanced prediction stability. Under ZIP scoring, where our method (MSE = 10.49 ± 0.47, RMSE = 3.24 ± 0.07, PCC = 0.86 ± 0.01) surpasses MGAE-DC by its 3.8% MSE. Comparing across method categories reveals a clear performance hierarchy: traditional machine learning approaches (SVR, Random Forest, XGBoost) exhibit substantially higher prediction errors depending on scoring scheme, representing 2.6× to 3× higher errors compared to our method. The narrow confidence intervals and low standard deviations observed for our method across all scoring schemes (standard deviations of 0.01–0.02 for PCC and 0.07–0.41 for RMSE) indicate robust performance across cross-validation folds, contrasting with the higher variance of traditional methods. Critically, the performance consistency across four distinct synergy metrics demonstrates that our model captures fundamental biological and pharmacological principles underlying drug combination effects rather than overfitting to specific quantification frameworks, with Pearson correlations ranging from 0.84 to 0.86

Table S1: Regression performance on the O'Neil dataset.

| Method | MSE | RMSE | CI | PCC |
|---|---|---|---|---|
| | | Loewe | | |
| SVR | 454.35 ± 33.74 | 21.30 ± 0.78 | [424.72, 483.97] | 0.35 ± 0.04 |
| Random Forest | 444.90 ± 19.40 | 21.09 ± 0.46 | [427.87, 461.93] | 0.39 ± 0.05 |
| XGBoost | 430.96 ± 20.81 | 20.75 ± 0.50 | [412.69, 449.23] | 0.40 ± 0.05 |
| Matchmaker | 394.42 ± 26.40 | 19.85 ± 0.66 | [371.24, 417.59] | 0.49 ± 0.06 |
| PRODeepSyn | 223.11 ± 25.56 | 14.91 ± 0.86 | [200.67, 245.54] | 0.76 ± 0.02 |
| MGAE-DC | <u>162.11 ± 17.03</u> | <u>12.72 ± 0.66</u> | [196.48, 299.55] | <u>0.83 ± 0.01</u> |
| HypertranSynergy | 185.61 ± 12.06 | 13.77 ± 0.07 | <u>[174.48, 212.39]</u> | 0.81 ± 0.02 |
| TensoGraph | **150.67 ± 10.07** | **12.27 ± 0.41** | **[141.83, 159.51]** | **0.84 ± 0.02** |
| | | Bliss | | |
| SVR | 41.91 ± 3.98 | 6.47 ± 0.31 | [38.41, 45.40] | 0.51 ± 0.02 |
| Random Forest | 41.38 ± 3.45 | 6.43 ± 0.27 | [38.36, 44.41] | 0.53 ± 0.02 |
| XGBoost | 40.87 ± 3.69 | 6.39 ± 0.29 | [37.63, 44.10] | 0.53 ± 0.02 |
| Matchmaker | 36.40 ± 3.42 | 6.03 ± 0.28 | [33.40, 39.41] | 0.60 ± 0.03 |
| PRODeepSyn | 24.15 ± 3.66 | 4.90 ± 0.37 | [20.94, 27.36] | 0.76 ± 0.03 |
| MGAE-DC | <u>17.05 ± 3.04</u> | <u>4.11 ± 0.36</u> | <u>[14.38, 19.72]</u> | <u>0.84 ± 0.02</u> |
| TensoGraph | **15.78 ± 3.23** | **3.95 ± 0.40** | **[13.47, 18.61]** | **0.85 ± 0.02** |
| | | HSA | | |
| SVR | 45.41 ± 5.29 | 6.73 ± 0.39 | [40.77, 50.05] | 0.44 ± 0.03 |
| Random Forest | 44.63 ± 3.73 | 6.67 ± 0.28 | [41.36, 47.90] | 0.46 ± 0.04 |
| XGBoost | 43.72 ± 4.25 | 6.60 ± 0.32 | [39.99, 47.45] | 0.47 ± 0.03 |
| Matchmaker | 39.51 ± 4.46 | 6.28 ± 0.36 | [35.60, 43.42] | 0.54 ± 0.05 |
| PRODeepSyn | 25.04 ± 4.13 | 4.99 ± 0.41 | [21.41, 28.67] | 0.75 ± 0.03 |
| MGAE-DC | <u>17.58 ± 2.05</u> | <u>4.19 ± 0.25</u> | <u>[15.79, 19.38]</u> | <u>0.83 ± 0.02</u> |
| TensoGraph | **15.70 ± 2.08** | **3.95 ± 0.26** | **[13.88, 17.53]** | **0.85 ± 0.01** |
| | | ZIP | | |
| SVR | 27.86 ± 1.08 | 5.28 ± 0.10 | [26.91, 28.81] | 0.53 ± 0.01 |
| Random Forest | 28.08 ± 0.73 | 5.30 ± 0.07 | [27.44, 28.72] | 0.53 ± 0.01 |
| XGBoost | 27.66 ± 1.07 | 5.26 ± 0.10 | [26.71, 28.60] | 0.53 ± 0.01 |
| Matchmaker | 25.18 ± 1.44 | 5.02 ± 0.14 | [23.91, 26.44] | 0.59 ± 0.04 |
| PRODeepSyn | 15.20 ± 0.77 | 3.90 ± 0.10 | [14.53, 15.88] | 0.78 ± 0.01 |
| MGAE-DC | <u>10.90 ± 0.49</u> | <u>3.30 ± 0.07</u> | <u>[10.47, 11.33]</u> | <u>0.85 ± 0.01</u> |
| TensoGraph | **10.49 ± 0.47** | **3.24 ± 0.07** | **[10.08, 10.91]** | **0.86 ± 0.01** |

validating accurate ranking of drug combinations by synergistic potential.

## 1.2 Regression Results on CLOUD dataset

Table S2 presents comprehensive regression performance metrics on the CLOUD dataset across seven prediction methods evaluated under four synergy scoring schemes (Loewe, Bliss, HSA, and ZIP), providing detailed quantitative validation of the performance trends illustrated in Figure 3 in the main text. The CLOUD dataset, characterized by broader drug class diversity and increased sample heterogeneity compared to O'Neil, serves as a critical benchmark for assessing model scalability and generalizability. Our proposed method TensoGraph demonstrates consistent superiority across all evaluation metrics and synergy frameworks, albeit with narrower performance margins reflecting the increased complexity of this dataset. Under Loewe additivity scoring, TensoGraph achieves MSE = 318.89 ± 15.93, RMSE = 17.85 ± 0.45, and PCC = 0.32 ± 0.03, with a 95% confidence interval of [304.91, 332.88], representing a 3.4% MSE improvement

Table S2: Regression performance on the CLOUD dataset.

| Method | MSE | RMSE | CI | PCC |
|---|---|---|---|---|
| | | Loewe | | |
| SVR | 359.85 ± 11.60 | 18.97 ± 0.30 | [349.67, 370.03] | 0.02 ± 0.02 |
| Random Forest | 341.47 ± 11.97 | 18.48 ± 0.32 | [330.96, 351.97] | 0.16 ± 0.03 |
| XGBoost | 341.01 ± 11.93 | 18.46 ± 0.32 | [330.54, 351.49] | 0.16 ± 0.03 |
| Matchmaker | 337.11 ± 12.19 | 18.36 ± 0.33 | [326.40, 347.81] | 0.20 ± 0.03 |
| PRODeepSyn | 398.48 ± 17.43 | 19.91 ± 0.52 | [378.29, 414.75] | 0.18 ± 0.03 |
| MGAE-DC | 330.12 ± 12.37 | 18.17 ± 0.34 | [319.25, 340.98] | 0.26 ± 0.03 |
| TensoGraph | **318.89 ± 15.93** | **17.85 ± 0.45** | **[304.91, 332.88]** | **0.32 ± 0.03** |
| | | Bliss | | |
| SVR | 366.14 ± 14.59 | 19.13 ± 0.38 | [353.34, 378.94] | 0.15 ± 0.02 |
| Random Forest | 343.00 ± 11.89 | 18.52 ± 0.32 | [332.57, 353.44] | 0.21 ± 0.02 |
| XGBoost | 342.53 ± 11.90 | 18.50 ± 0.32 | [332.09, 352.98] | 0.21 ± 0.02 |
| Matchmaker | 344.98 ± 12.16 | 18.57 ± 0.33 | [334.30, 355.65] | 0.24 ± 0.03 |
| PRODeepSyn | 396.52 ± 20.77 | 19.91 ± 0.52 | [378.29, 414.75] | 0.18 ± 0.03 |
| MGAE-DC | 327.94 ± 11.23 | 18.11 ± 0.31 | [318.08, 337.80] | 0.31 ± 0.02 |
| TensoGraph | **319.00 ± 9.75** | **17.86 ± 0.27** | **[310.44, 327.56]** | **0.36 ± 0.02** |
| | | HSA | | |
| SVR | 359.81 ± 10.54 | 18.97 ± 0.28 | [350.56, 369.06] | 0.02 ± 0.02 |
| Random Forest | 341.35 ± 7.55 | 18.48 ± 0.20 | [334.72, 347.98] | 0.16 ± 0.04 |
| XGBoost | 341.00 ± 7.56 | 18.47 ± 0.20 | [334.37, 347.64] | 0.16 ± 0.04 |
| Matchmaker | 338.23 ± 7.62 | 18.39 ± 0.21 | [331.54, 344.91] | 0.19 ± 0.03 |
| PRODeepSyn | 396.10 ± 11.40 | 19.90 ± 0.29 | [386.10, 406.11] | 0.13 ± 0.03 |
| MGAE-DC | 321.14 ± 9.43 | 17.92 ± 0.26 | [312.86, 329.42] | 0.29 ± 0.04 |
| TensoGraph | **316.92 ± 11.59** | **17.80 ± 0.32** | **[306.74, 327.10]** | **0.32 ± 0.04** |
| | | ZIP | | |
| SVR | 366.44 ± 22.62 | 19.13 ± 0.59 | [346.58, 386.30] | 0.15 ± 0.03 |
| Random Forest | 343.03 ± 18.87 | 18.51 ± 0.51 | [326.47, 359.59] | 0.21 ± 0.02 |
| XGBoost | 342.71 ± 18.89 | 18.51 ± 0.51 | [326.13, 359.29] | 0.21 ± 0.02 |
| Matchmaker | 341.49 ± 18.46 | 18.47 ± 0.50 | [325.28, 357.70] | 0.23 ± 0.02 |
| PRODeepSyn | 395.40 ± 18.97 | 19.88 ± 0.48 | [378.75, 412.06] | 0.19 ± 0.02 |
| MGAE-DC | 327.72 ± 18.52 | 18.10 ± 0.51 | [311.46, 343.97] | 0.31 ± 0.02 |
| TensoGraph | **320.33 ± 20.07** | **17.89 ± 0.57** | **[302.72, 337.95]** | **0.36 ± 0.02** |

over the second-best method MGAE-DC (MSE = 330.12 ± 12.37, PCC = 0.26 ± 0.03). The PCC improvement from 0.26 to 0.32 represents a 23% relative increase in correlation strength, demonstrating enhanced capture of synergistic patterns. Under Bliss independence scoring, our method outperform MGAE-DC by 2.7% in MSE and showing a 16% relative PCC improvement. For HSA scoring, TensoGraph achieves a 1.3% MSE reduction compared to the second best, while under ZIP scoring, our method shows a 2.3% MSE improvement over the second best MGAE-DC. Traditional machine learning methods exhibit substantially degraded PCC performance on this more complex dataset. This suggest that learned representations are particularly advantageous for capturing complex synergistic mechanisms such as synthetic lethality and compensatory pathway inhibition in datasets with increased heterogeneity.

## 1.3 Regression Results on ALMANAC dataset

Table S3 presents comprehensive regression performance metrics on the ALMANAC dataset across seven prediction methods evaluated under four synergy scoring schemes (Loewe, Bliss,

Table S3: Regression performance on the ALMANAC dataset.

| Method | MSE | RMSE | CI | PCC |
|---|---|---|---|---|
| | | Loewe | | |
| SVR | $>10^5$ | $>10^5$ | — | $>10^5$ |
| Random Forest | 266.61 ± 4.86 | 16.33 ± 0.15 | [262.34, 270.88] | 0.23 ± 0.01 |
| XGBoost | 259.69 ± 4.98 | 16.11 ± 0.15 | [255.32, 264.06] | 0.25 ± 0.01 |
| Matchmaker | 252.63 ± 5.52 | 15.89 ± 0.17 | [247.78, 257.48] | 0.31 ± 0.03 |
| PRODeepSyn | 194.60 ± 34.15 | 13.90 ± 1.17 | [164.62, 224.58] | 0.62 ± 0.04 |
| MGAE-DC | 121.18 ± 1.92 | 11.01 ± 0.09 | [118.11, 124.26] | 0.75 ± 0.01 |
| TensoGraph | **115.48 ± 3.22** | **10.75 ± 0.15** | **[112.65, 118.31]** | **0.77 ± 0.01** |
| | | Bliss | | |
| SVR | $>10^5$ | $>10^5$ | — | $>10^5$ |
| Random Forest | 32.23 ± 0.64 | 5.68 ± 0.06 | [31.67, 32.80] | 0.23 ± 0.02 |
| XGBoost | 31.16 ± 0.66 | 5.58 ± 0.06 | [30.58, 31.74] | 0.27 ± 0.02 |
| Matchmaker | 30.66 ± 0.80 | 5.54 ± 0.07 | [29.96, 31.37] | 0.30 ± 0.02 |
| PRODeepSyn | 28.37 ± 4.63 | 5.31 ± 0.41 | [24.30, 32.44] | 0.56 ± 0.03 |
| MGAE-DC | 15.89 ± 0.29 | 3.99 ± 0.04 | [15.63, 16.15] | 0.73 ± 0.01 |
| TensoGraph | **14.80 ± 0.37** | **3.85 ± 0.05** | **[14.48, 15.13]** | **0.75 ± 0.01** |
| | | HSA | | |
| SVR | $>10^5$ | $>10^5$ | — | $>10^5$ |
| Random Forest | 28.74± 0.51 | 5.36 ± 0.05 | [28.29, 29.19] | 0.32 ± 0.01 |
| XGBoost | 28.13 ± 0.46 | 5.30 ± 0.04 | [27.73, 28.53] | 0.34 ± 0.01 |
| Matchmaker | 27.28 ± 0.67 | 5.22 ± 0.06 | [26.69, 27.87] | 0.38 ± 0.03 |
| PRODeepSyn | 28.27 ± 4.74 | 5.30 ± 0.45 | [24.11, 32.44] | 0.60 ± 0.04 |
| MGAE-DC | 12.97 ± 0.23 | 3.60 ± 0.03 | [12.77, 13.17] | **0.77 ± 0.01** |
| TensoGraph | **12.01 ± 0.44** | **3.47 ± 0.06** | **[11.63, 12.39]** | **0.77 ± 0.01** |
| | | ZIP | | |
| SVR | $>10^5$ | $>10^5$ | — | $>10^5$ |
| Random Forest | 24.71 ± 0.44 | 4.97 ± 0.04 | [24.32, 25.10] | 0.40 ± 0.01 |
| XGBoost | 24.62 ± 0.47 | 4.96 ± 0.05 | [24.21, 25.04] | 0.40 ± 0.01 |
| Matchmaker | 24.48 ± 0.53 | 4.95 ± 0.05 | [24.01, 24.94] | 0.42 ± 0.02 |
| PRODeepSyn | 23.28 ± 3.83 | 4.81 ± 0.40 | [19.92, 26.64] | 0.60 ± 0.03 |
| MGAE-DC | 12.97 ± 0.23 | 3.60 ± 0.03 | [12.69, 13.07] | 0.77 ± 0.01 |
| TensoGraph | **11.93 ± 0.24** | **3.45 ± 0.04** | **[11.71, 12.14]** | **0.79 ± 0.01** |

HSA, and ZIP), providing detailed quantitative validation of performance trends illustrated in Figure 4 in the main text. The ALMANAC dataset, comprising extensive combinatorial screening data across a large panel of diverse cancer cell lines and drug modalities, represents the most challenging evaluation scenario due to its scale and inherent heterogeneity. Notably, SVR fails to converge due to computational constraints and data complexity, highlighting the limitations of traditional machine learning on large-scale screening problems. Our method TensoGraph demonstrates robust superiority across all metrics and scoring frameworks. Under Loewe additivity scoring, TensoGraph achieves MSE = 115.48 ± 3.22, RMSE = 10.75 ± 0.15, and PCC = 0.77 ± 0.01, with a 95% confidence interval of [112.65, 118.31], representing a 4.7% MSE improvement over the second-best method MGAE-DC (MSE = 121.18 ± 1.92, PCC = 0.75 ± 0.01). For Bliss independence, our method attains MSE = 14.80 ± 0.37, RMSE = 3.85 ± 0.05, and PCC = 0.75 ± 0.01, outperforming MGAE-DC (MSE = 15.89 ± 0.29, PCC = 0.73 ± 0.01) by 6.9% in MSE. Under HSA scoring, TensoGraph achieves a 4.6% MSE reduction compared to MGAE-DC. Most substantially, under ZIP scoring, our method achieves the lowest

prediction error with a 7.8% MSE improvement over MGAE-DC. Traditional machine learning methods exhibit catastrophic failure or severe degradation on this large-scale dataset. Among deep learning approaches, PRODeepSyn shows substantially higher variance, while MGAE-DC consistently maintains second-best performance. The significantly narrower confidence intervals and lower standard deviations for our method across all metrics (e.g., CI width of 5.66 for Loewe versus 9.15 for MGAE-DC) indicate enhanced stability and generalizability on large-scale heterogeneous data. The consistent performance across all four synergy metrics (PCC ranging from 0.75 to 0.79) validates the model's robustness in capturing subtle interaction patterns influenced by complex mechanisms such as pathway redundancy and dynamic cellular rewiring. These results establish the model's efficacy for real-world large-scale drug discovery applications where identifying effective combinations is critical to overcoming resistance and improving therapeutic indices.

# 2 Performance Evaluation on Specific Cell Lines

## 2.1 Performance analysis of O'Neil across all synergy metrics

Figure S1 provides additional information on the Loewe and ZIP synergy scoring schemes that are not presented in the main text. We incorporate all four synergy scoring schemes into the analysis of the O'Neill dataset. The analysis reveals consistent performance patterns across all metrics, establishing the robustness of our approach. Under Loewe additivity (Figures S1(a) and 1(e)), OURS maintains PCC values predominantly above 0.8 across 33 out of 39 cell lines, with RMSE remaining below 15 in 33 cell lines, demonstrating exceptional stability on this stringent additivity metric. For ZIP scoring (Figures S1(d) and S1(h)), our method achieves similarly superior performance with PCC values exceeding 0.8 in 31 cell lines and RMSE consistently below 4 across nearly all cell lines, indicating enhanced prediction reliability on this zero-interaction potency framework. The cross-metric consistency observed across Figures S1(a-d) and S1(e-h) is particularly noteworthy: for individual cell lines, the performance ranking of methods remains highly consistent regardless of the synergy scoring scheme employed. This metric-agnostic performance consistency suggests that our model has learned generalizable interaction patterns that transcend specific mathematical formulations of synergy, rather than merely fitting to particular scoring schemes. Comparing the four synergy metrics on a per-method basis reveals that our method demonstrates the highest performance stability, with minimal PCC variance across metrics. Our method shows remarkably smooth curves across cell lines within each metric, while other approaches exhibit dramatic fluctuations, particularly for challenging cell lines.

## 2.2 Performance analysis of ALMANAC across all synergy metrics

To assess the robustness and generalizability of the proposed framework, we extended our evaluation to the ALMANAC dataset, which comprises 39 cell lines from the NCI-60 cancer

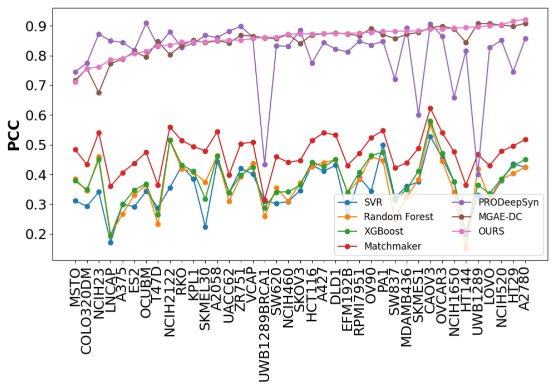


(a) O'Neil Loewe - PCC

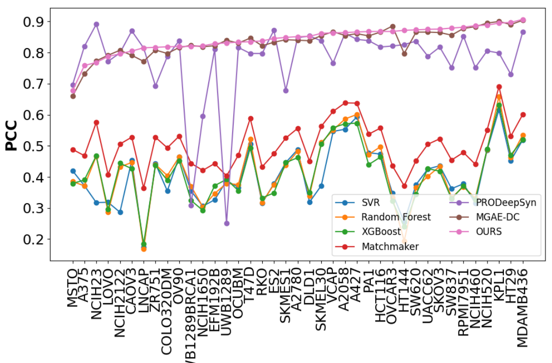


(b) O'Neil Bliss - PCC

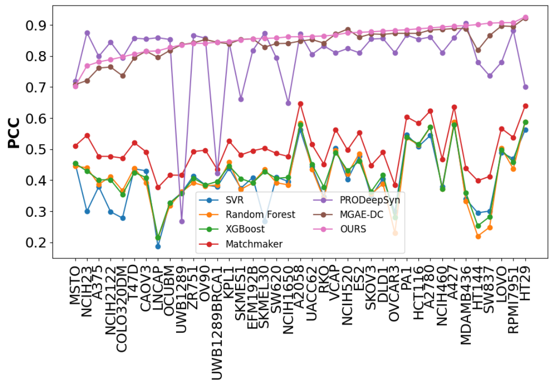


(c) O'Neil HSA - PCC

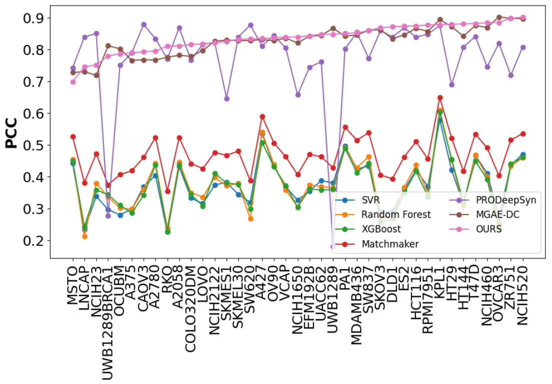


(d) O'Neil ZIP - PCC

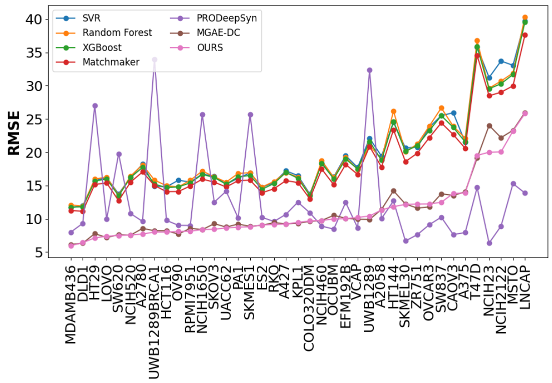


(e) O'Neil Loewe - RMSE

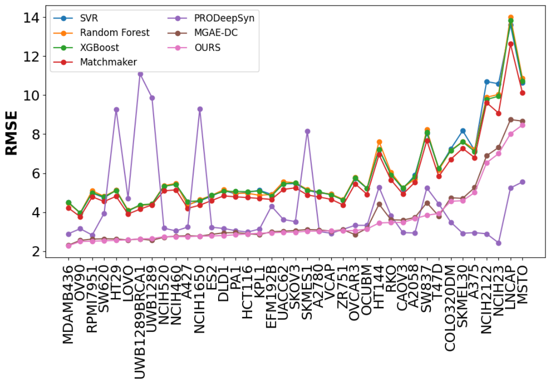


(f) O'Neil Bliss - RMSE

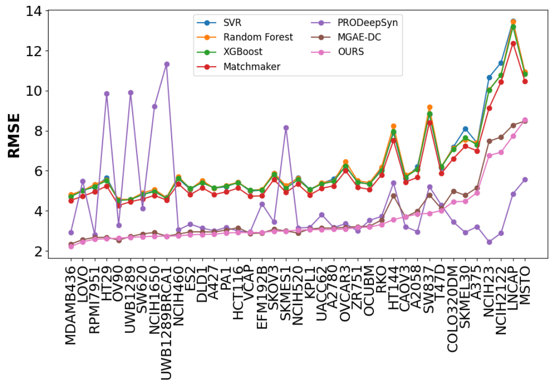


(g) O'Neil HSA - RMSE

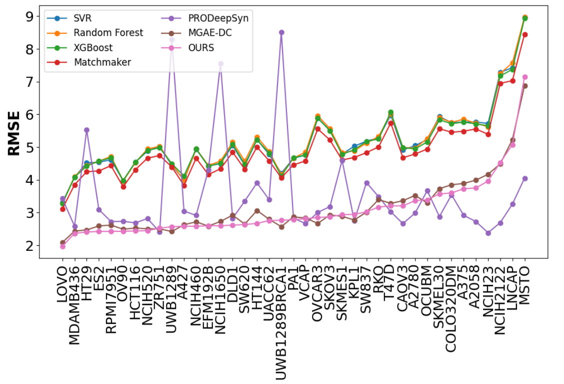


(h) O'Neil ZIP - RMSE

Figure S1: Cell line-specific performance comparison of drug synergy prediction methods in the O'Neil dataset. This figure presents the predictive performance of seven methods including SVR, Random Forest, XGBoost, Matchmaker, PRODeepSyn, MGAE-DC, and our model across all 39 cell lines, showing Pearson correlation coefficient values in (a)-(d) and RMSE values in (e)-(h), highlighting cell line-specific differences in prediction accuracy.

panel. Comprehensive comparisons across four synergy scoring schemes—Bliss, HSA, Loewe, and ZIP—demonstrate consistent advantages of deep learning–based methods (PRODeepSyn, MGAE-DC, and OURS) over traditional machine learning baselines. As shown in Figure S2, OURS achieves the highest predictive accuracy across nearly all cell lines, with PCC values consistently above 0.7, and outperforms the second-best model (MGAE-DC) in almost every case.

The RMSE distributions further reveal that conventional methods suffer from large prediction errors and unstable performance across heterogeneous genetic backgrounds. In contrast, the smooth RMSE curves observed for OURS and other deep learning models indicate stable and generalizable predictions. S2 shows that TensoGraph maintains superior performance with reduced variance across all 39 cell lines. Collectively, these results establish the model's robust generalization across multiple mathematical formulations of drug synergy and highlight its capacity to capture biologically consistent patterns in large-scale pharmacogenomic data.

## 2.3 Cell line-specific embedding analysis

An essential validation of our model is to assess whether it captures biologically meaningful and cell line-specific interaction patterns from drug combination synergy data. To investigate this, we focused on a representative compound, BEZ-235 (a dual PI3K/mTOR inhibitor), and extracted its cell-specific interaction features learned by the model across all 39 cell lines in the O'Neil dataset. We applied t-distributed stochastic neighbor embedding (t-SNE) to reduce the high-dimensional drug-cell line interaction embeddings to two dimensions for visualization. As illustrated in Figure S3, each point represents a cell line, with colors indicating tissue of origin across six major cancer types: melanoma, colon, ovarian, lung, breast, and prostate. The visualization reveals clear clustering of cell lines by tissue type. This tissue-specific clustering pattern demonstrates that our model effectively captures pharmacogenomic signatures that are inherently linked to cellular lineage and molecular context. The learned embeddings encode not merely drug chemical properties or isolated cell line features, but rather context-dependent interaction patterns that reflect how specific cellular backgrounds modulate drug combination responses.

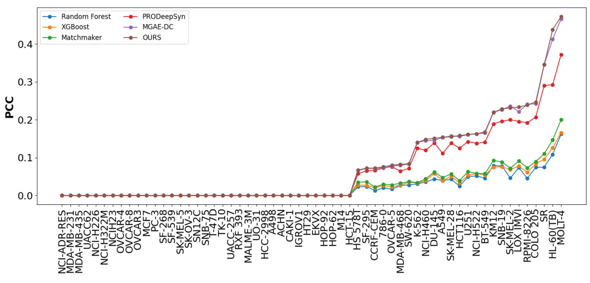


(a) ALMANAC Loewe - PCC

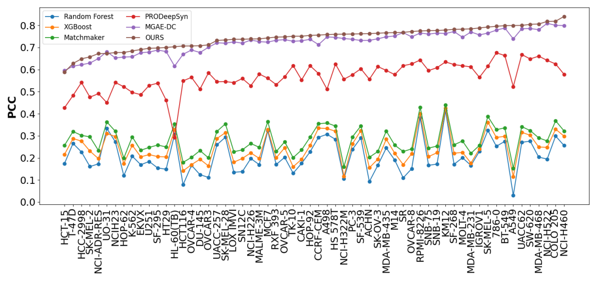


(b) ALMANAC Bliss - PCC

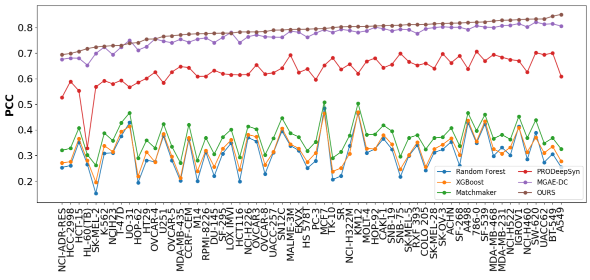


(c) ALMANAC HSA - PCC

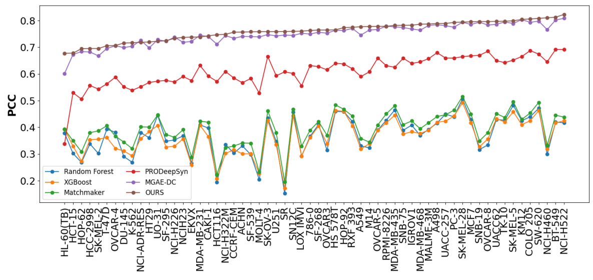


(d) ALMANAC ZIP - PCC

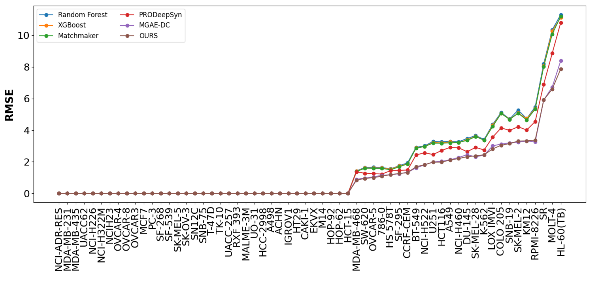


(e) ALMANAC Loewe - RMSE

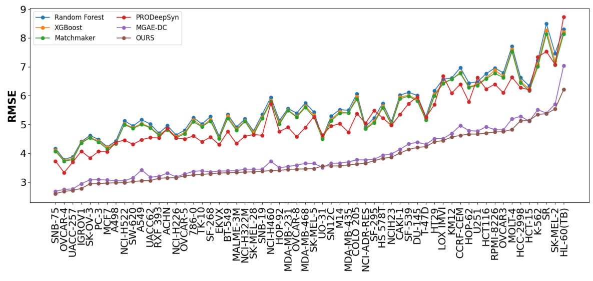


(f) ALMANAC Bliss - RMSE

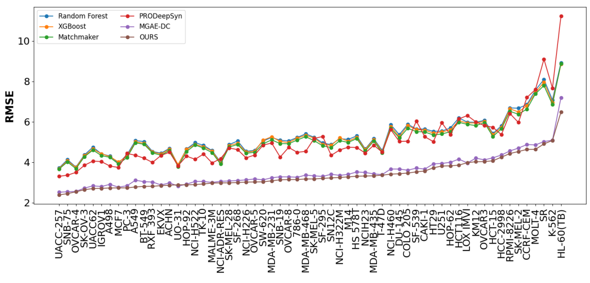


(g) ALMANAC HSA - RMSE

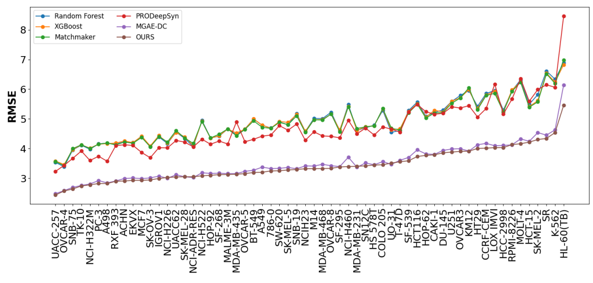


(h) ALMANAC ZIP - RMSE

Figure S2: Cell line-specific performance comparison of drug synergy prediction methods in the ALMANAC dataset. This figure presents the predictive performance of the same seven methods across all 59 cell lines, displaying PCC values in (a)-(d) and RMSE values in (e)-(h), emphasizing the variation of prediction accuracy across individual cell lines.

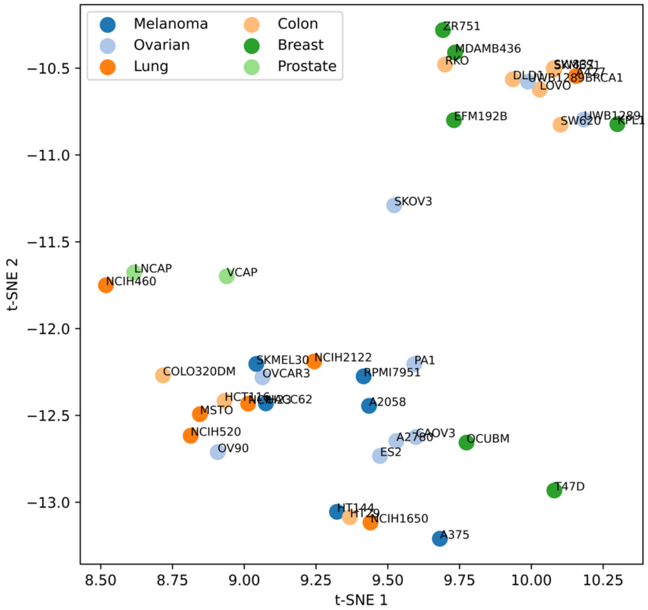


Figure S3: Visualization of cell line specific drug embeddings on the O'Neil dataset. Two-dimensional visualization of the cell line specific embeddings of the BEZ-235 using t-distributed stochastic neighbor embedding (t-SNE) on the O'Neil dataset. Each point corresponds to a cell line, with colors indicating six tissue origins including melanoma, colon, ovarian, lung, breast, and prostate cancers.

# 3 Detailed analysis of representative cell lines

Figure S4 presents comprehensive comparisons for the O'Neil dataset across three representative cell lines—MDAMB436, KPL1, and LNCAP—which respectively correspond to cases with the best, moderate, and relatively poorer predictive performance. The scatter plots in the left columns of Figures S4(a)–(b) show strong linear correlations between predicted and observed synergy scores under both Loewe and Bliss independence scoring, with regression lines closely following the ideal $y = x$ relationship. The middle and right columns summarize RMSE and PCC distributions across all methods, demonstrating that OURS consistently achieves among the lowest RMSE and highest PCC values. Even for the challenging LNCAP cell line (androgen-sensitive prostate cancer), OURS maintains reasonable predictive accuracy (PCC $= 0.82$), substantially outperforming traditional machine learning approaches (PCC $< 0.4$). Figures S4(c)–(d) further supplement the performance under HSA and ZIP scoring schemes, where OURS continues to exhibit excellent regression fit with slopes of 1.01–1.06 and PCC values of 0.84–0.86 across the three cell lines, consistently surpassing traditional methods (PCC $< 0.5$). The tight regression fits and low variance across metrics confirm the robust generalization capability of our learned representations.

Figures S5 supplements the synergy scoring schemes omitted from Figure 6 in the main text. Figures S5(a) and S5(b) present the ALMANAC results under Loewe and Bliss scoring schemes.

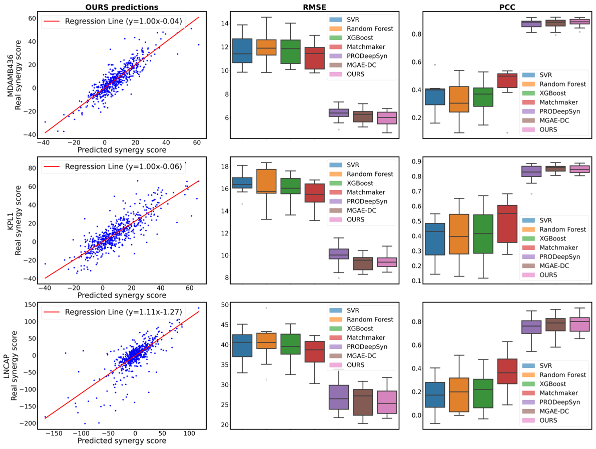


(a) O'Neil Loewe

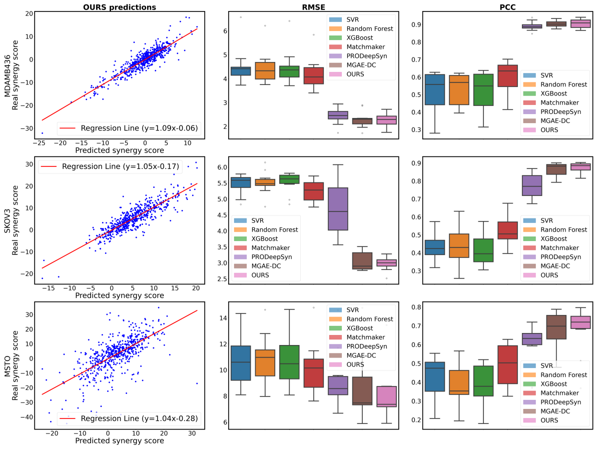


(b) O'Neil Bliss

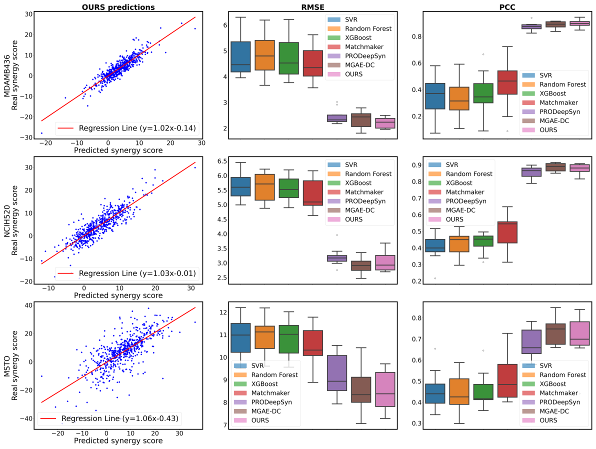


(c) O'Neil HSA

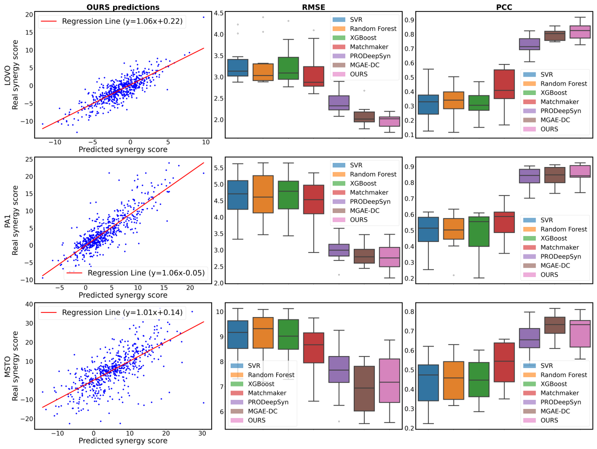


(d) O'Neil ZIP

Figure S4: Representative cell line performance for drug synergy prediction in the O'Neil dataset. Comparative results are shown for three selected cell lines under four synergy scoring schemes including Loewe, Bliss, HSA, and ZIP, with figures illustrating regression between predicted and observed synergy scores on the left, RMSE comparison in the middle, and PCC comparison on the right, highlighting model robustness on representative cases.

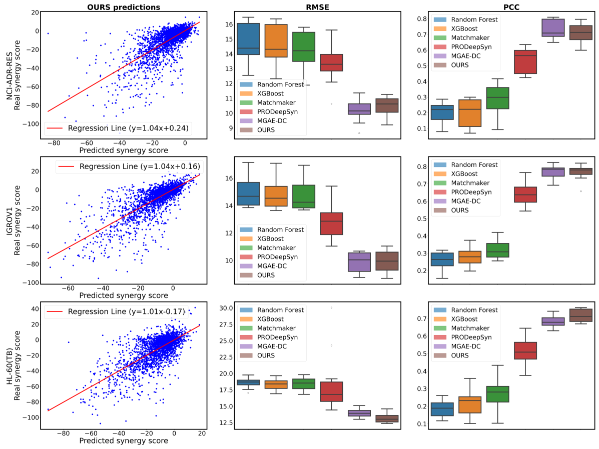


(a) ALMANAC Loewe

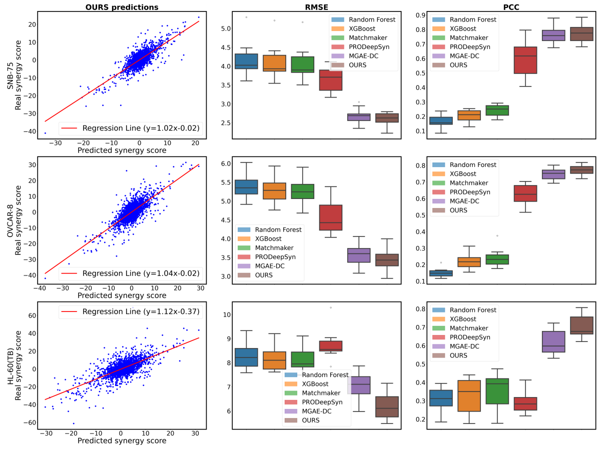


(b) ALMANAC Bliss

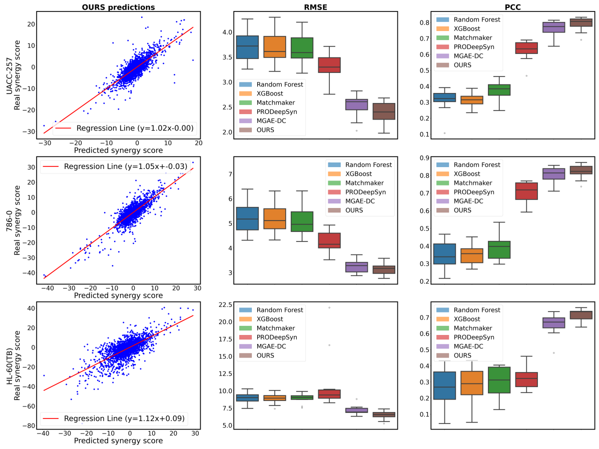


(c) ALMANAC HSA

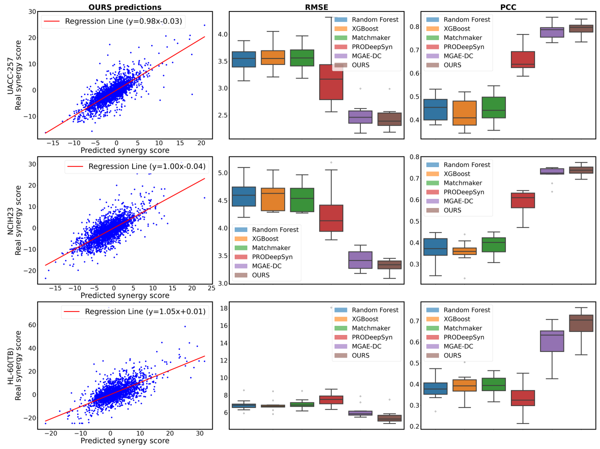


(d) ALMANAC ZIP

Figure S5: Representative cell line performance for drug synergy prediction in the ALMANAC dataset. Comparative results are shown for three selected cell lines with figures illustrating regression between predicted and observed synergy scores on the left, RMSE comparison in the middle, and PCC comparison on the right, demonstrating prediction consistency and model reliability on representative cell lines.

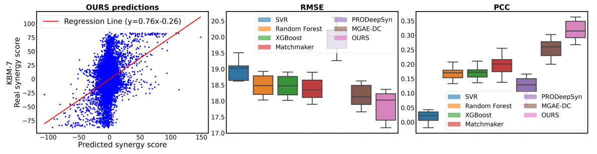


(a) CLOUD Loewe

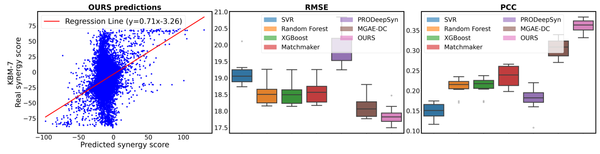


(b) CLOUD Bliss

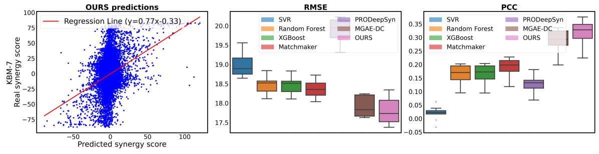


(c) CLOUD HSA

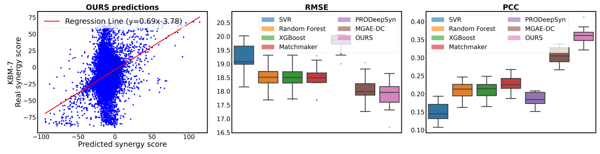


(d) CLOUD ZIP

Figure S6: Representative cell line performance for drug synergy prediction in the CLOUD dataset. This figure shows results for a representative cell line, with regression between predicted and observed synergy scores on the left, RMSE comparison in the middle, and PCC comparison on the right, illustrating the predictive performance of the seven methods on a complex dataset with high drug diversity.

Across all four ALMANAC metrics, our method consistently achieves tighter regression fits and lower prediction variance compared to baseline methods, validating robust generalization on large-scale heterogeneous data.

Figure S6 presents the comparative performance for CLOUD across all four synergy scoring schemes (Loewe, Bliss, HSA, ZIP) using a representative cell lines. Although the performance of all methods has significantly declined on the CLOUD dataset, the predicted slope of our method still matches the actual slope, ranging from 0.69 to 0.77. Compared to baseline methods, our approach maintains substantially tighter regression fits and lower prediction variance, with traditional machine learning methods exhibiting substantially degraded performance and wide error distributions. These results on CLOUD corroborate the robustness of our method across datasets of varying scales and compositions, confirming its applicability to diverse real-world drug screening scenarios.

# 4 Ablation Study Details

We systematically construct six model configurations following a carefully designed exclusion hierarchy to isolate the contribution of each component:

- **OURS w/o A&Tucker&GTN:** This represents the most minimal baseline configuration, utilizing only pre-computed drug molecular fingerprints and cell line genomic features (gene expression profiles). This variant serves as the reference baseline to measure the aggregate contribution of all graph-based and tensor-based learning components.

- **OURS w/o A&Tucker:** This variant incorporates GTN-based local drug interaction features while excluding both GCN molecular graph encodings and Tucker decomposition global features. It relies on drug fingerprints, cell line features, and GTN-derived pairwise interaction embeddings. This configuration specifically evaluates the contribution of local graph transformer-based relational learning in the absence of molecular structural encoding and global tensor factorization.

- **OURS w/o A&GTN:** This configuration excludes both GCN structural encodings and GTN local features, retaining only Tucker decomposition-derived global interaction patterns combined with fingerprint and cell line features. This design isolates the contribution of tensor factorization in capturing multi-way drug-drug-cell interactions without explicit molecular graph or local transformer-based representations.

- **OURS w/o Tucker&GTN:** This variant removes all drug interaction modeling components (both local GTN features and global Tucker features), limiting the framework to GCN-encoded molecular graph representations, drug fingerprints, and cell line genomic features. This setup evaluates the sufficiency of molecular structure encoding alone for synergy prediction without explicit drug-drug interaction modeling.

- **OURS w/o A:** This configuration excludes only the GCN-based molecular graph encoding component, retaining all interaction modeling mechanisms including GTN local features, Tucker global features, drug fingerprints, and cell line features. This variant assesses the necessity of explicit molecular graph representation when comprehensive interaction features are available.

- **OURS (TensoGraph):** The complete architecture integrating all components—GCN molecular graph encoding, GTN local interaction features, Tucker decomposition global features, drug fingerprints, and cell line genomic profiles—providing the comprehensive framework for drug synergy prediction.

## 5 Parameter Sensitivity Analysis

Model hyperparameters critically influence the trade-off between expressiveness and generalizability in drug synergy prediction. To quantify this relationship, we conducted a grid search on the O'Neil–Loewe dataset, varying three key parameters: (1) the embedding dimension $d$ of the graph transformer networks (GTN), (2) the number of aggregation channels $C$ in GTN, and (3) the embedding size of the downstream MLP layers. This analysis provides empirical guidance for model configuration and reveals how architectural capacity shapes biological pattern learning.

As shown in Figure S7, performance is most sensitive to the GTN embedding dimension $d$. Optimal accuracy is achieved at $d$=32, which balances representational richness with computational efficiency. Nearby values of $d$ yield comparable results, reflecting the model's robustness to moderate dimensional perturbations.

Varying the channel width $C$ shows diminishing returns: increasing $C$ marginally improves performance but substantially raises computational cost. Hence, $C$=2 is selected as a balanced configuration for all experiments.

Finally, the downstream MLP embedding dimension governs the expressiveness of the predictive layers. Performance improves with larger embeddings but plateaus beyond a certain threshold, indicating overparameterization. An embedding size of 8192 achieves stable, high-fidelity predictions without excessive model complexity.

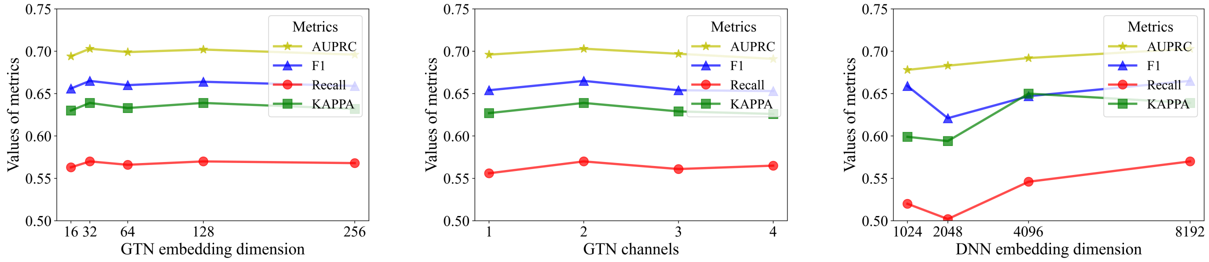


Figure S7: Impact of model hyperparameters on drug synergy prediction. We evaluates the effect of GTN embedding dimensions $d$, number of graph channels $C$, and DNN embedding dimensions on the O'Neil dataset using the Loewe synergy score, showing how different parameter settings influence the predictive performance of our model across diverse cell lines and drug combinations.
